# Genome-wide identification and functional characterization of the ABF gene family in maize reveals ZmABF8 as a key regulator of drought tolerance

**DOI:** 10.3389/fpls.2026.1849922

**Published:** 2026-05-21

**Authors:** Zixu Guo, Xuechun Gao, Chaoqun Wang, Zhibo Liu, Ziqi Chen, Shunlan Ning, Mengchao Xiao, Jianbo Fei, Qi Zhang

**Affiliations:** 1 College of Agriculture, Jilin Agricultural University, Changchun, China; 2 College of Agriculture, Jilin Agricultural Science and Technology College, Jilin, China; 3 Institute of Agricultural Biotechnology, Jilin Academy of Agricultural Sciences (Northeast Innovation Center of Agricultural Science and Technology in China), Changchun, China

**Keywords:** ABF gene family, bioinformatics, Maize (Zea mays L.), phylogenetic analysis, stress response

## Abstract

Abscisic acid (ABA) plays a pivotal role in plant adaptation to abiotic stresses, with ABA-responsive element binding factors (ABFs) serving as central regulators in ABA signaling pathways. Despite their importance, the ABF gene family in maize (*Zea mays L.*) remains poorly characterized. Here, we performed the first comprehensive genome-wide analysis of the maize ABF gene family, identifying 18 *ZmABF* members distributed across 9 chromosomes. Phylogenetic analysis classified these genes into four distinct subgroups, all containing conserved bZIP domains and predicted nuclear localization. Promoter analysis revealed abundant ABA-responsive cis-elements, particularly ABRE and ARE motifs, suggesting strong ABA-mediated regulation. Expression profiling demonstrated tissue-specific patterns, with predominant expression in roots and leaves, and significant upregulation under drought, cold, and heat stresses. Notably, protein-protein interaction analysis revealed that all ZmABFs interact with SnRK2 kinases, confirming their integration into the core ABA signaling network. Functional validation focused on *ZmABF8*, which showed the most pronounced induction under drought stress. Overexpression of *ZmABF8* in maize significantly enhanced drought tolerance, as evidenced by improved seed germination, reduced seedling damage, and increased activities of antioxidant enzymes (POD and SOD) under water deficit conditions. These findings not only provide a systematic genomic resource for the maize ABF family but also identify *ZmABF8* as a promising genetic target for developing drought-resistant maize varieties in the face of climate change.

## 1 Introduction

Maize is an important food crop and industrial raw material worldwide, and it is highly susceptible to abiotic stresses during production, leading to yield losses. Statistics indicate that extreme drought conditions can lead to a 58% reduction in maize yield, with a 60% decrease in pollen viability during the pollination period (He et al., 2024). High temperature and low temperature stress are also significant factors contributing to the reduction of corn yield (Liu et al., 2024a). Therefore, enhancing the tolerance of corn to adverse stress is crucial for ensuring corn production.

The ABF gene is an important member of the bZIP transcription factor family in plants and is a core component of the ABA signaling pathway (Bensmihen, 2005). It plays a crucial regulatory role during seed germination, dormancy, and early seedling development (Zinsmeister et al., 2016). Studies have also shown that this gene family plays a significant role in responses to abiotic stress (Waadt et al., 2022). The proteins encoded by this gene family contain a conserved basic leucine zipper (bZIP) domain, which is associated with protein-protein interactions and DNA binding activity. This domain primarily interacts with the ACGT cis-acting element in target gene promoters, and also includes TAGTTA (A-box), GACGTC (C-box), and CACGTG (G-box) (Bensmihen et al., 2002).

Nine ABF family members were identified in Arabidopsis, while 23 were found in Brassica, and 8 and 9 were identified in chickpea and lentil, respectively (Yoshida et al., 2010; Bai et al., 2016; Basso et al., 2025). Studies have shown that during the seedling stage of Arabidopsis, the ABF subfamily members exhibit differential response patterns to treatments such as ABA, high salinity, osmotic stress, and low temperature (Sun et al., 2025). Among these, *ABF1* and *ABI5* are the most sensitive to ABA and osmotic stress, with 20 μmol/L ABA treatment resulting in nearly a 30-fold increase in their mRNA relative content; treatment with 100 mmol/L sorbitol further upregulated the expression of *ABF1* and *ABI5* by approximately 120-fold and 30-fold, respectively. For salt stress, *ABF3* showed the most significant upregulation; under 4 °C low-temperature treatment, the expression of ABF1 and ABI5 increased by approximately 110-fold and 25-fold, respectively (Wang et al., 2021). In chickpea and lentil, the AREB/ABF/ABI5 subfamily genes exhibited significant upregulation under PEG-simulated drought stress, and higher expression levels of these genes were observed in varieties with stronger drought resistance, indicating that they could serve as potential targets for crop stress resistance breeding (Ayub et al., 2025). Similarly, in different ratoon years of sugarcane varieties, ABF was identified as an ABA-related differentially expressed gene, which may be closely related to the important agronomic trait of ratoon ability (Collin et al., 2021).

The function of ABF in tolerance to abiotic stress relies on a precise molecular regulatory network, and different types of stress may trigger distinct response mechanisms of ABF (Skubacz et al., 2016). Under drought stress, ABF enhances crop drought tolerance by regulating the expression of ABA-induced genes such as LEA proteins (Fujita et al., 2011); under salt stress, its expression is induced by salt stress, directly or indirectly regulating genes related to osmoprotectants and ion homeostasis (De et al., 2021); under high-temperature stress, it participates in heat stress response by regulating key genes related to chlorophyll degradation (such as PPH, PAO) and interacting with proteins like MYB44 (Liu et al., 2023); while under low-temperature stress, ABF mediates low-temperature signaling pathways by interacting with low-temperature response transcription factors such as ICE1 (Hu et al., 2019). Despite extensive research on the mechanisms of ABF family gene functions in different crops, most of the current understanding is derived from studies in Arabidopsis, with few reports in maize (Finkelstein and Lynch, 2000). Although the ABF family genes are relatively conserved, there are still significant differences among different crops. Therefore, it is essential to conduct identification and functional analysis of ABF family genes in maize.

This study conducts a comprehensive genome-wide identification of the maize ABF gene family and performs extensive bioinformatics analyses, including physicochemical property analysis of proteins, gene structure analysis, synteny analysis, protein-protein interaction analysis, analysis of cis-acting elements in promoters, and expression pattern analysis. Furthermore, systematic functional validation of the *ZmABF8* gene, which positively responds to drought stress, was carried out. The results indicate that this gene significantly enhances maize tolerance under drought stress, providing important theoretical support for further elucidating the biological functions of the ABF gene family.

## 2 Results

### 2.1 Identification of ZmABF gene family members in maize

By performing a BLAST comparison with the protein sequences of the ABF gene family in *Arabidopsis thaliana* and combining it with HMM identification, 18 members of the maize ABF gene family were identified, located on 9 out of 10 chromosomes. Based on their positions, these genes have been renamed as *ZmABF1-ZmABF18*. By systematically analyzing the basic characteristics of the 18 members of the *ZmABF* gene family, it was found that the number of amino acid residues in ZmABF family members ranges from 161 (*ZmABF9*) to 412 (*ZmABF17*). The number of amino acid residues in ZmABF17 reaches a maximum of 412, which shows a difference of 251 amino acid residues compared to the minimum *ZmABF9*. The molecular weight range of family members spans from 17874.22 (*ZmABF9*) to 43446.87 (*ZmABF17*). The isoelectric point (pI) parameters fluctuate between 5.44 (*ZmABF6*) and 9.98 (*ZmABF13*). The instability index of the proteins shows significant variation (39.98-83.06), with particular attention drawn to *ZmABF10*, which has the lowest instability index (39.98). The aliphatic index exhibits significant variation, ranging from 39.98 to 78.88. The hydrophilic parameter GRAVY values are all negative (-0.851 to -0.379), indicating that all members are hydrophilic proteins. Subcellular localization predictions suggest that the ZmABF family is expressed in the nucleus (**Supplementary Table 1**).

### 2.2 *ZmABF* gene structure and conserved domain analysis

**Figure 1:**
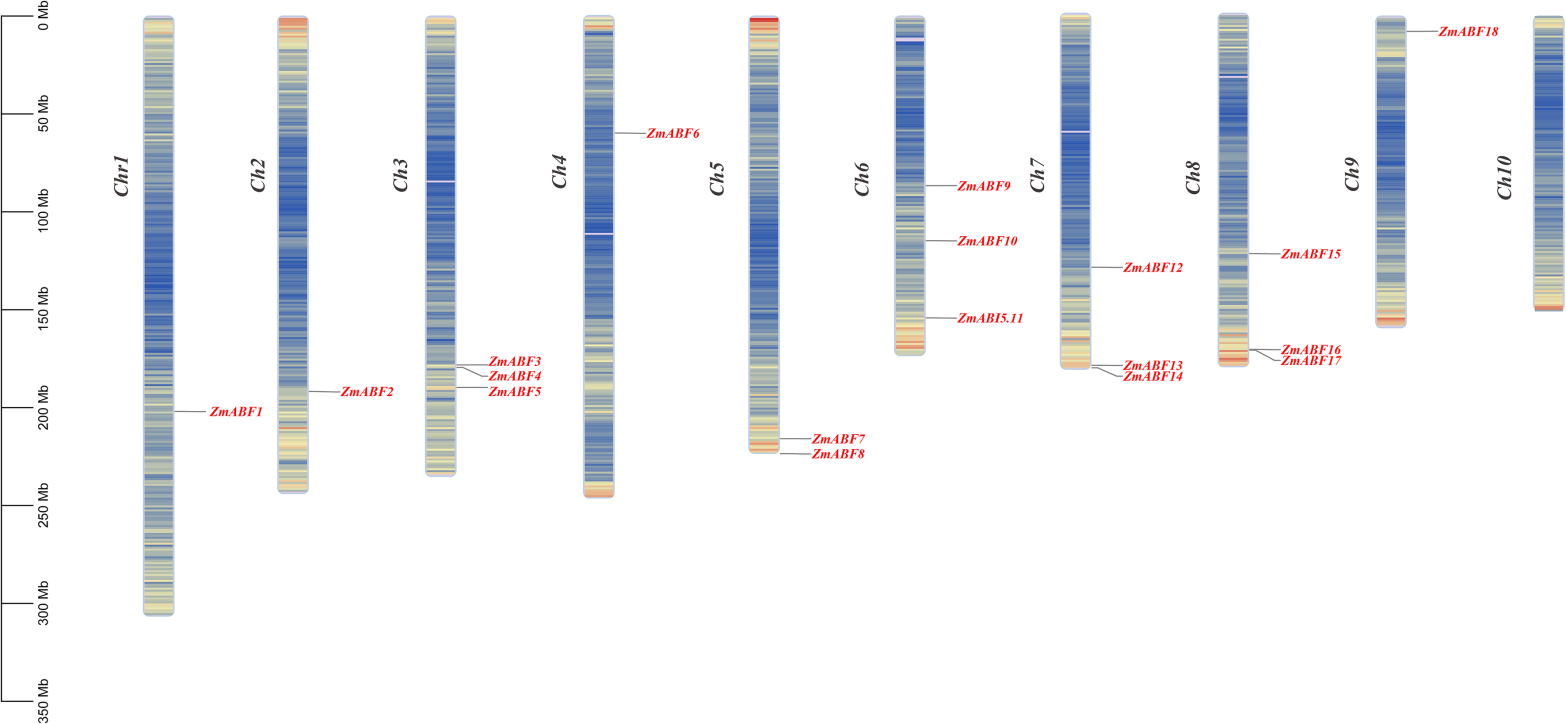
Chromosomal localization analysis of the *ZmABF* gene in maize. The left side indicates the chromosome number, while the right side corresponds to the gene name. A color gradient from red to blue on the chromosome represents the trend of gene density variation from high to low, where darker areas signify regions with higher gene density, and lighter areas indicate regions with lower gene density. Unmarked regions on the chromosome denote genetic segments lacking gene distribution data.

**Figure 2:**
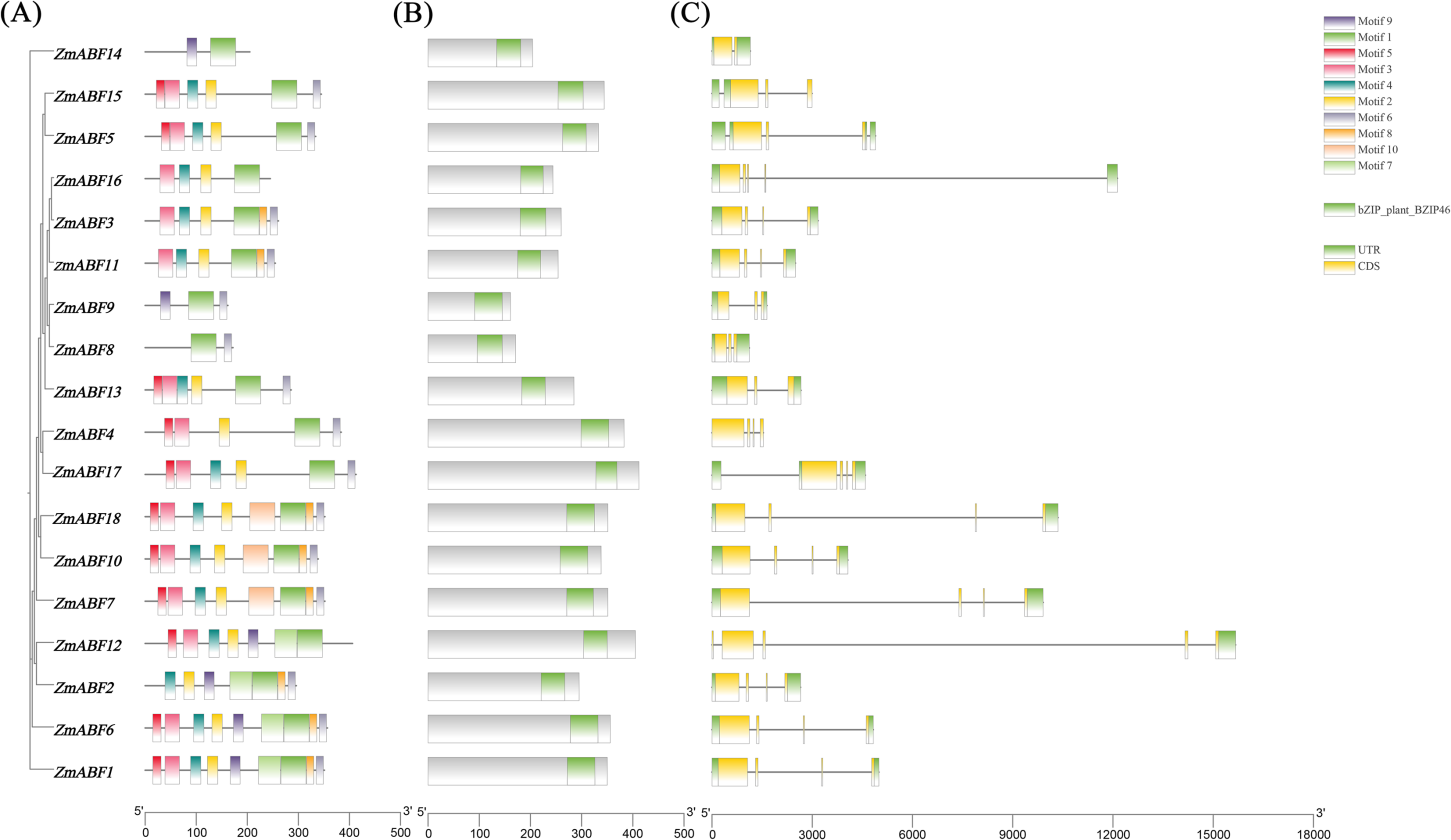
Conserved motifs, domains, and pattern of the *ZmABF* gene in maize. **(A)** Conserved domains of ZmABF. Different colored boxes represent distinct conserved domains identified in each protein, and their positions are shown according to the protein sequence length. **(B)** Conserved motif composition of ZmABF. Each colored box represents a specific conserved motif predicted by MEME, and the arrangement of motifs along the protein sequences is illustrated. **(C)** Gene structure of ZmABF, including exons, introns, and untranslated regions (UTRs). Exons, introns, and UTRs are indicated by boxes and lines with different colors, and their positions are displayed relative to the genomic sequence from 5′ to 3′.

The 18 members of the *ZmABF* gene family in maize are distributed across 9 chromosomes (**Figure 1**). Chromosomes 1, 2, 4, and 9 each contain one *ZmABF* gene, while chromosomes 3, 6, 7, and 8 each contain three members, and chromosome 5 contains two members. This characteristic of cross-chromosomal dispersed distribution reflects the multiple duplication and differentiation events that this gene family may have undergone during the course of evolution. The analysis of the structure, conserved domains, and motifs of the *ZmABF* gene reveals its functional differentiation and evolutionary characteristics. A total of 10 characteristic motifs were identified in this gene family, among which motif 1 is highly conserved across all subfamilies, indicating its critical role in maintaining the core function of the ZmABF protein (**Figure 2A**; **Supplementary Figure 1**). All members of the *ZmABF* gene family carry the conserved domain of the bZIP superfamily, confirming the reliability of the phylogenetic classification of this family (**Figure 2B**). The gene structure of *ZmABF* exhibits significant heterogeneity, with the number of exons ranging from 2 (as in *ZmABF14*) to 5 (as in *ZmABF12*). *ZmABF4* lacks both the 5’ UTR and 3’ UTR, suggesting that it has a less complex post-transcriptional regulatory mechanism compared to other members (**Figure 2C**).

### 2.3 *ZmABF* gene phylogenetic and synteny analysis

**Figure 3:**
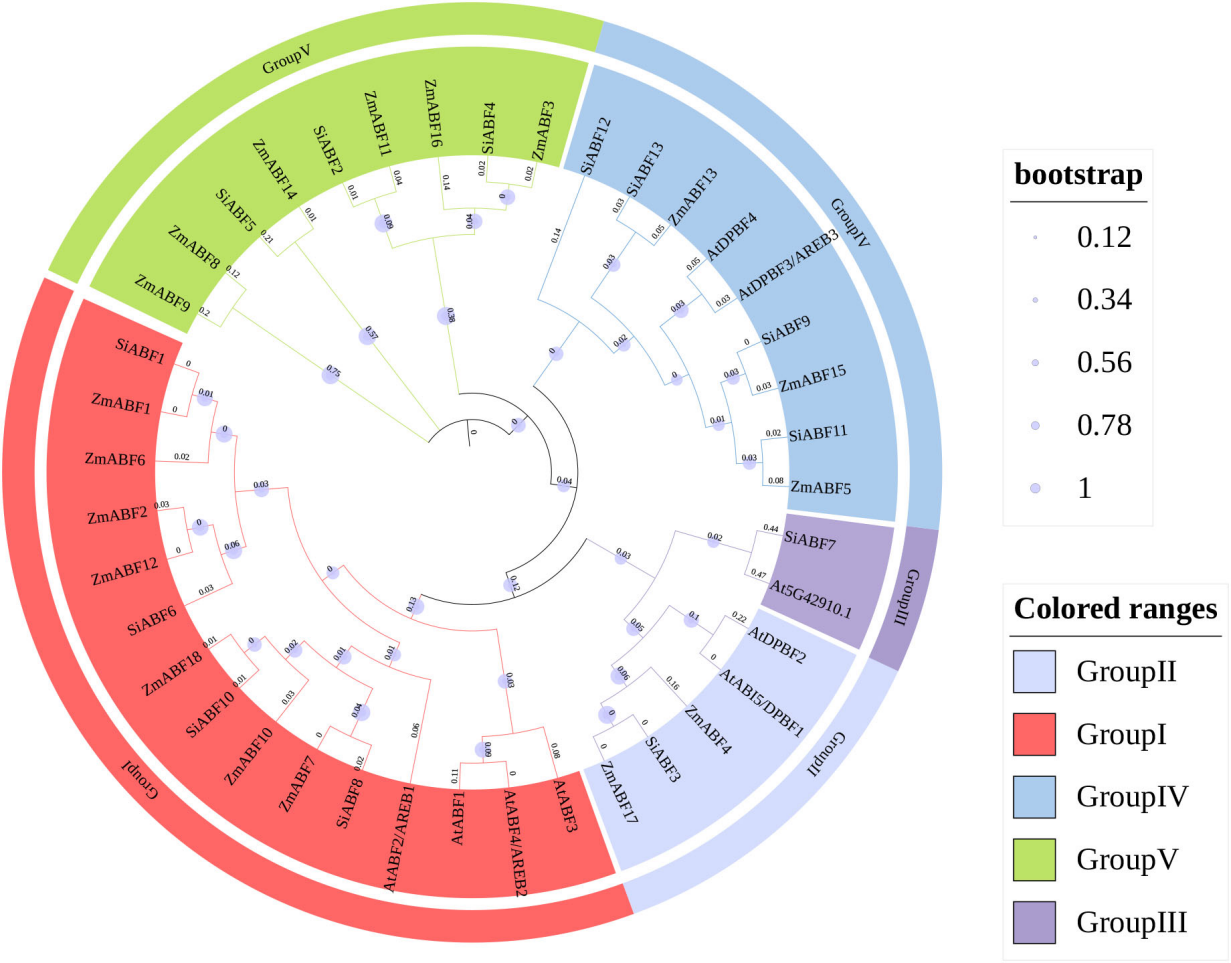
Phylogenetic tree of ABF in maize, millet, and Arabidopsis. Arabidopsis thaliana. The unrooted phylogenetic tree was constructed using the Maximum Likelihood (ML) method. Different species are distinguished by their gene name prefixes: ‘Zm’ represents maize (*Zea mays*), ‘Si’ represents foxtail millet (Setaria italica), and ‘At’ represents Arabidopsis thaliana. The outer colored rings and branch colors indicate the five distinct phylogenetic subgroups (Group I to Group V). The light purple circles on the branch nodes represent bootstrap values (based on 1000 replicates), with the size of the circles indicating the level of bootstrap confidence.

**Figure 4:**
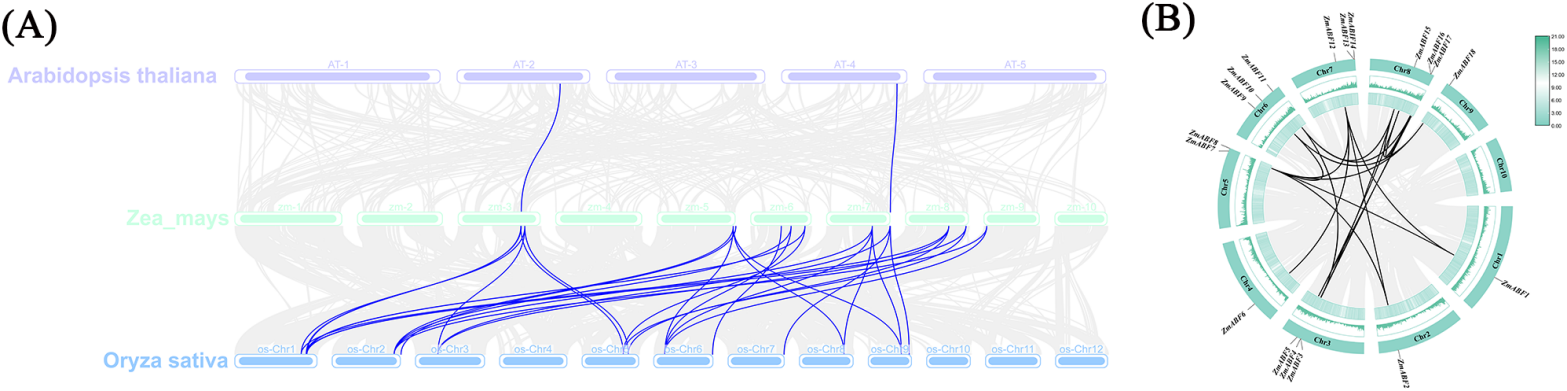
Collinearity analysis of *ZmABF* gene family. **(A)** Inter species collinearity analysis of maize *ZmABF* gene family with Arabidopsis and rice. **(B)** Intraspecific collinearity analysis of *ZmABF* gene family in maize.

This study constructed a phylogenetic tree based on the amino acid sequences of the *ABF* gene from millet, rice, and *Arabidopsis thaliana*. All branch nodes of the phylogenetic tree exhibit significantly high confidence. According to the classic classification framework of the *AtABF* family in Arabidopsis, all ABF proteins can be divided into five subgroups (Group I–V). However, in the phylogenetic tree constructed in this study, the *ZmABF* members of maize were only distributed in four branches, namely Group I, II, IV and V, and no members belonging to Group III were detected (**Figure 3**). This phenomenon may be related to the lineage-specific loss or functional differentiation of this branch during the evolution of grass plants. Group I and Group V contain 7 and 6 *ZmABF* members respectively, and are the two largest branches in the maize *ABF* family.The *ZmABF* gene from maize and the SiABF gene from millet form a highly supported branch, indicating the evolutionary conservation of the ABF gene among Poaceae crops. Analysis of gene collinearity among maize, rice, and *Arabidopsis thaliana*. The results indicate that there are 29 pairs of ABF homologous gene pairs between maize and rice, located on chromosomes 3, 5, 6, 7, 8, and 9 of maize (**Figure 4A**). We used the Simple Ka/Ks Calculator in TBtools to evaluate the synonymous (Ks) and non-synonymous (Ka) substitution rates of the duplicated gene pairs. The results indicated that the Ka/Ks ratios of all maize ABF gene pairs were less than 1, ranging from 0.25 to 0.61, suggesting that these genes have undergone strong purifying selection during evolution. Additionally, the variations in Ks values indicated that the duplication events occurred at different evolutionary periods (**Supplementary Table 5**).

### 2.4 ZmABF gene interaction network and regulatory elements analysis

**Figure 5:**
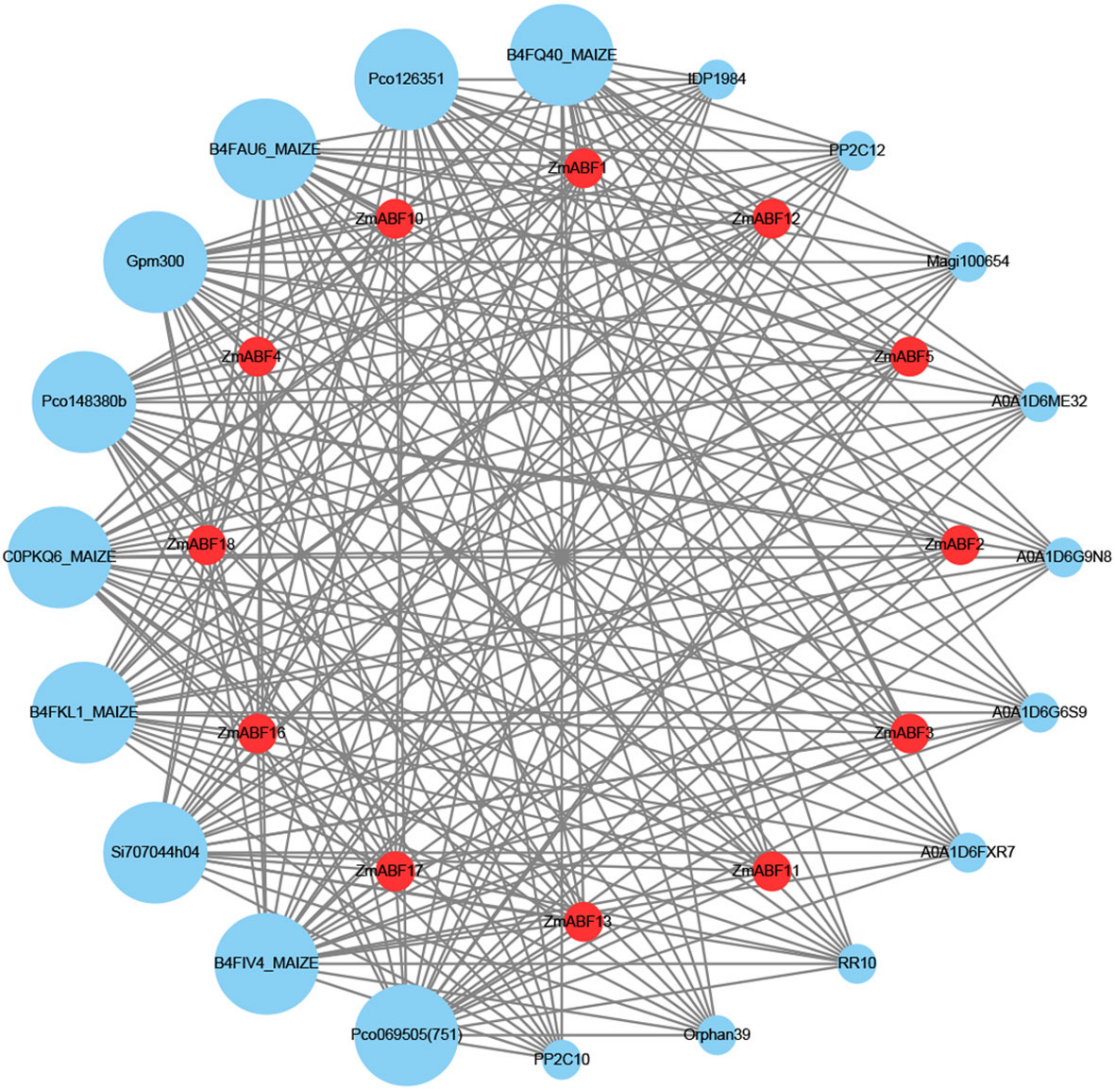
ABF gene interaction network analysis. The ZmABF protein is represented by red nodes, while the remaining proteins are depicted using blue nodes. The names or families of all interacting proteins are labeled according to the annotation rules of the STRING database. The size of the nodes reflects the number of interaction relationships that each protein participates in; larger nodes indicate a higher number of interacting proteins. The lines represent the interactive associations between proteins, with the thickness of the lines corresponding to the confidence level of the interaction; thicker lines indicate a higher level of confidence in the interaction.

To investigate the potential interactions of the ZmABF protein, we screened public data for proteins that are reliably associated with ZmABF (≥0.8). Based on this, we constructed a protein interaction network. In this network, the names of the ZmABF gene family proteins are self-named. The names of other interacting proteins or their respective family names are presented according to the annotation style of the STRING database. The predicted results indicate that the proteins interacting with ZmABF all belong to the serine/threonine protein kinase family (SnRK2s) (**Figure 5**). ZmABF serves as a core hub in the abscisic acid signaling pathway. ABF primarily regulates plant responses to abiotic stresses such as drought, high salinity, and low temperatures. During the transcriptional regulatory process of the ZmABF transcription factor, it is likely that the phosphorylation by SnRK2s plays a stabilizing role for the ZmABF protein, helping it to maintain its function and enhance resistance to abiotic stresses.

**Figure 6:**
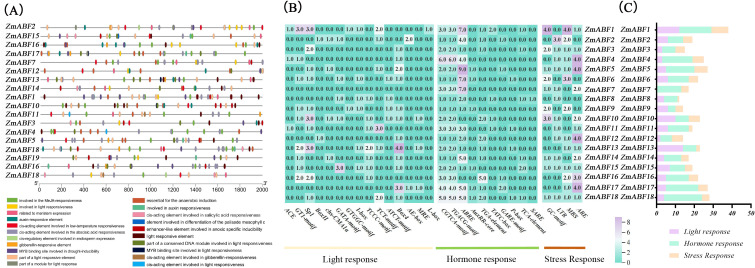
Analysis of the cis-regulatory elements of the maize *ZmABF* gene. **(A)** Spatial distribution pattern of cis-acting elements in the promoter region of the *ZmABF* gene. **(B, C)** Quantitative statistics of functional elements in the promoter region of the *ZmABF* gene: yellow corresponds to light-responsive elements, green represents hormone-responsive elements, and brown indicates environmental stress-related elements.

To gain a deeper understanding of the functional characteristics of the *ZmABF* gene family, we predicted the cis-regulatory elements within 2000 bp upstream of the promoter sequences of its members, and visualized the analysis results through bioinformatics methods. A total of 48 types of cis-acting elements were predicted, among which 15 are light-responsive elements led by ACE, GT1-motif, and Box II; 10 are hormone-responsive elements primarily characterized by ABRE, TATC-box, and P-box. Four types of stress response elements primarily involving ARE and MBS, along with 19 cis-acting elements related to other functions, have been identified (**Figure 6**). Among the 18 identified *ZmABF* genes, 10, 16, and 14 genes respectively contain Sp1, ABRE, and ARE elements. The 18 cis-regulatory elements of *ZmABF* are associated with light response, salicylic acid response, auxin response, low-temperature response, drought induction, and hypoxia-specific induction. These analytical results indicate that the ABF gene family in maize may possess dual regulatory functions: it not only participates in activating the plant’s stress response (including both biotic and abiotic stresses) but may also be involved in the signaling processes of plant hormones such as auxins.

**Figure 7:**
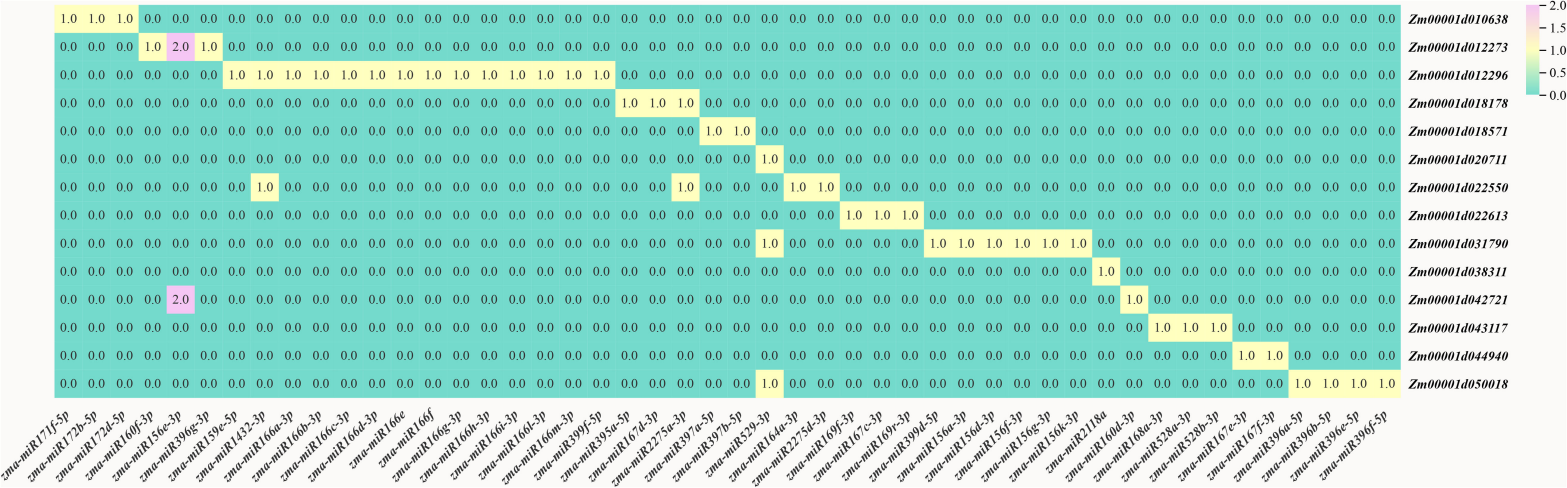
*ZmABF* gene miRNA prediction.

MicroRNAs (miRNAs) are non-coding small RNAs derived from eukaryotes and serve as crucial post-transcriptional regulatory factors. They primarily regulate the translation process by binding to the mRNA of target genes, thereby becoming an important means of post-transcriptional regulation of target gene expression. Based on the previous analysis of the target gene structure, 18 identified genes, of which 17 possess a 5’ untranslated region (5’ UTR) (**Figure 7**). Through predictions and analyses in bioinformatics, specific miRNA binding sites have been identified within the 5’ UTR regions of these genes (*ZmABF15, ZmABF16, ZmABF17, ZmABF7, ZmABF8, ZmABF12, ZmABF3, ZmABF14, ZmABF1, ZmABF11, ZmABF3, ZmABF5, ZmABF18 and ZmABF6*). Although all 14 genes have corresponding miRNAs that bind to their 5’ UTR regions, only the *ZmABF15* gene directly inhibits translation upon binding with miR171c (**Supplementary Table 2**). The other genes’ associated miRNAs block gene expression through non-translational repression mechanisms, such as promoting mRNA degradation or interfering with ribosome assembly.

### 2.5 *ZmABF* gene expression patterns analysis

**Figure 8:**
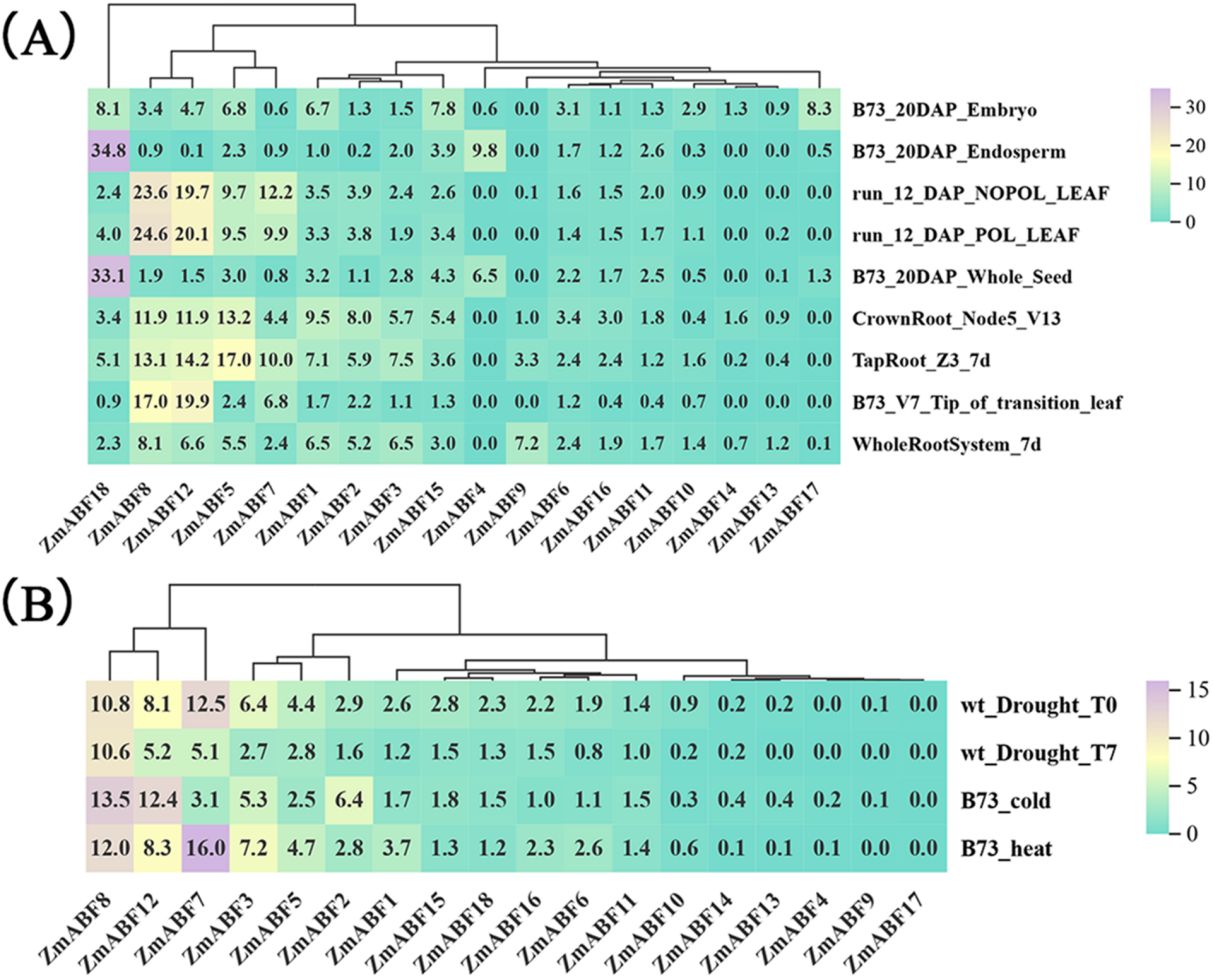
*ZmABF* expression pattern analysis based on RNA-seq data. **(A)** Expression levels of *ZmABF* genes in various tissue parts of maize at different growth stages. **(B)** Expression level analysis of *ZmABF* genes under drought, cold and heat stress.

To elucidate the biological functions of the *ZmABF* gene family, this study systematically analyzes the spatiotemporal expression characteristics of its members in various tissues and organs of maize, based on resources from the public MaizeGDB expression database. Focusing on the adaptive responses of nutritional organs (roots, leaves) and the regulatory mechanisms of reproductive organ development (seeds), a gene expression heatmap visualization analysis revealed that *ZmABF18* is highly expressed in the endosperm, embryo, and seed tissues (**Figure 8**). *ZmABF1*, *ZmABF2*, *ZmABF3*, *ZmABF5, ZmABF6, ZmABF7, ZmABF8, ZmABF10, ZmABF11, ZmABF12, ZmABF15, ZmABF16, and ZmABF18* are expressed in all tissues, and all genes are also expressed in B73_20DAP_Embryo and WholeRootSystem_7d (**Figure 8A**). There is a significant differentiation in expression patterns between the primary root and leaf tissues. Certain genes exhibit higher expression levels in the embryo and endosperm tissues, while their expression profiles differ in nutritional organ tissues such as roots and leaves. Additionally, different members of the family show varying expression within the same tissue, suggesting that members of the maize ABF gene family may play diversified regulatory roles in various physiological processes such as embryo development, endosperm formation, and root-leaf growth. This expression specificity provides a transcriptional basis for their functional differentiation.

By integrating preliminary research data and conducting a whole-genome RNA-seq analysis of maize, this study systematically explores the response patterns of the *ZmABF* gene family to drought, cold, and heat stresses (**Figure 8B**). The experimental treatment system includes: drought stress (wt_Drought_T0, wt_Drought_T7) and cold and heat treatments (B73_cold, B73_heat). Heatmap analysis based on expression profiles indicates that the *ZmABF* gene family in maize exhibits significant response characteristics to drought stress, with most family members showing differential expression patterns under drought conditions. Especially, *ZmABF3, ZmABF5, ZmABF7*, *ZmABF8*, and *ZmABF12* exhibited positive responses to various abiotic stresses. Among them, *ZmABF8* exhibits extremely high transcriptional activity under both drought stress and low temperature treatment, indicating that it may play a significant role in plant stress resistance.

**Figure 9:**
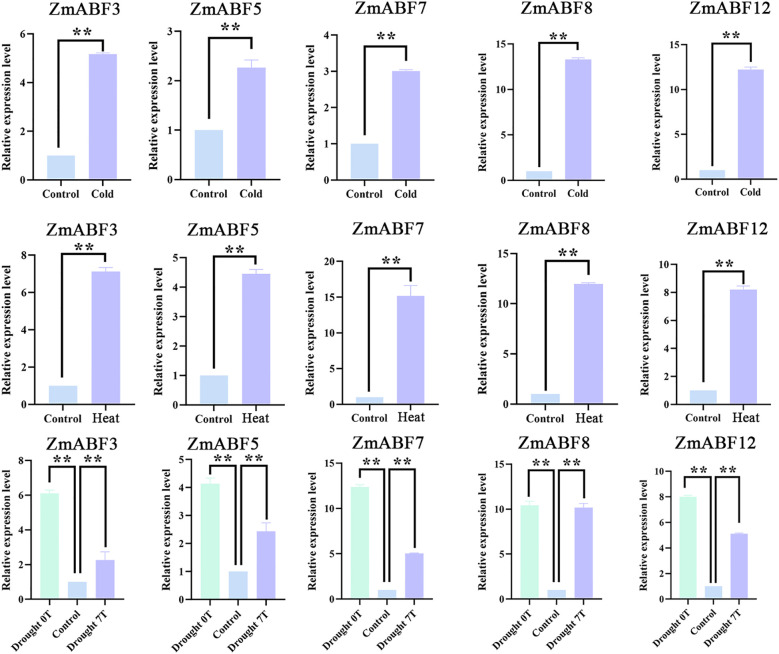
*ZmABF* gene in heat, cold and drought stress response expression pattern analysis based on qPCR. Data are shown as mean ± SD from three independent experiments. 0T represents the expression level after 0 days of stress. 7T represents the gene expression level after 7 days of drought stress.

This study chose *ZmABF3, ZmABF5, ZmABF7, ZmABF8*, and *ZmABF12* for qPCR expression validation. The results are shown in **Figure 9**. Under cold stress conditions, the expression levels of the five genes increased by 2 to 10 times compared to the control, with all increases being highly significant. Under heat stress, the expression levels of the five genes increased by more than six times compared to the control. This result is consistent with the RNA-seq analysis results. In addition, we subjected these five genes to drought stress and conducted expression analysis by sampling on the day of stress and on the seventh day of stress. The results indicated that the expression levels of all five genes significantly increased on the day of stress (**Figure 9**). This is related to the involvement of this gene family in ABA regulation. ABA responds rapidly and sensitively to drought and is activated at the onset of drought. The *ZmABF* gene family exhibited high expression immediately after the onset of drought, indicating a close regulatory relationship between this gene family and ABA. Notably, the expression level of the *ZmABF8* gene was significantly elevated by more than 10-fold compared to the control under cold, heat, and drought stress. Especially under drought stress, this gene not only responded rapidly but also maintained a high expression level until the seventh day of stress. This gene is highly representative of *ZmABF* in responding to abiotic stress, especially drought stress.

### 2.6 *ZmABF* gene expression patterns analysis

**Figure 10:**
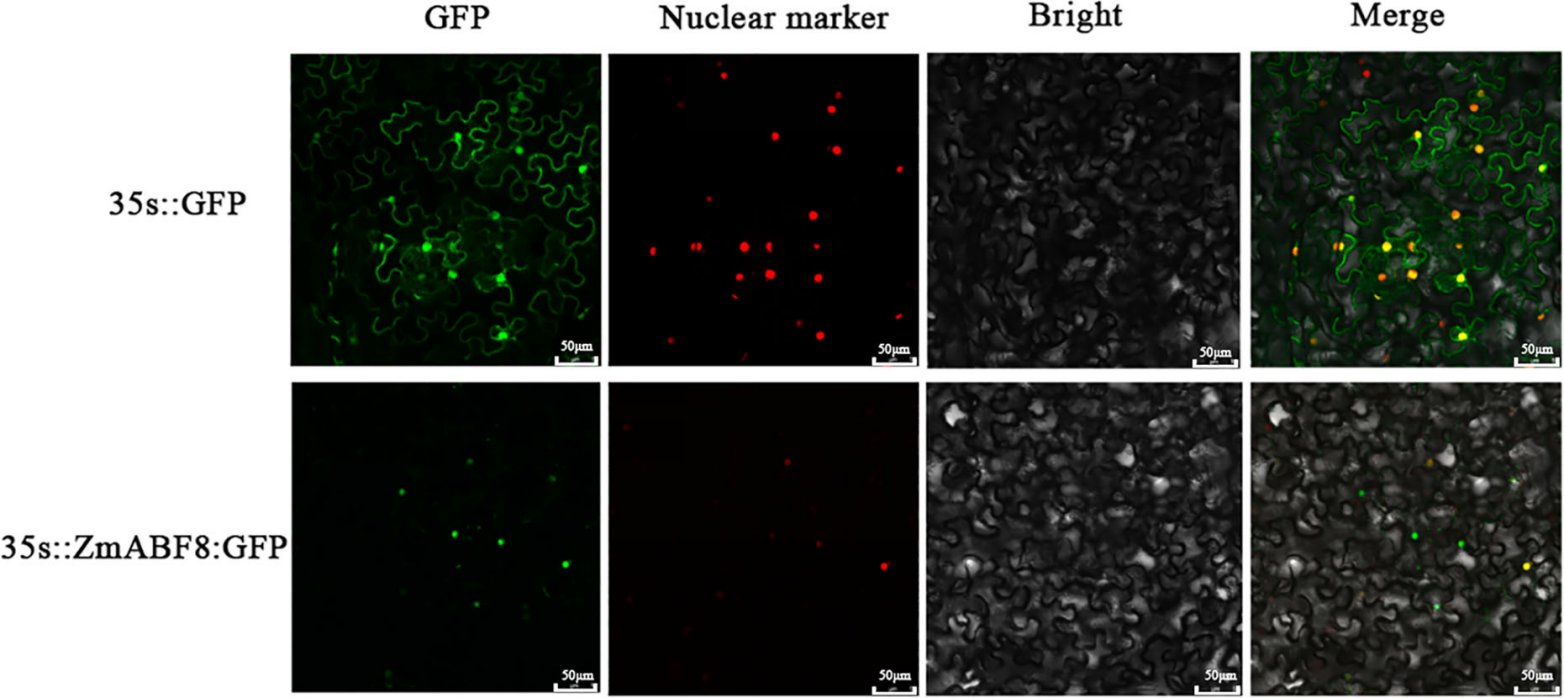
Subcellular localization of *ZmABF8*. *ZmABF8*-green fluorescent protein (GFP) recombinant and pCambia1302GFP control vector were transiently expressed in tobacco mesophyll cells (Scale bars = 50 μm). Red fluorescent protein (RFP) is the nuclear localization signal (Nuclear marker).

Based on the characteristics of *ZmABF8*’s active response to abiotic stress within the *ZmABF* gene family, this study investigates the subcellular localization of *ZmABF8*, using transient expression in *Nicotiana benthamiana* as a representative method. **Figure 10** clearly shows that the *ZmABF8*-GFP fusion protein is specifically localized in the nucleus, while the pCambia1302-GFP control is distributed throughout the entire cell. These results indicate that the *ZmABF8*-GFP fusion protein has a distinct nuclear localization, suggesting that it may interact with other nuclear proteins or participate in the regulation of gene expression within the nucleus (**Figure 10**). Understanding this subcellular localization is crucial for elucidating the functions and behaviors of members of the *ZmABF* gene family.

### 2.7 *ZmABF8* phenotype analysis under drought stress

**Figure 11:**
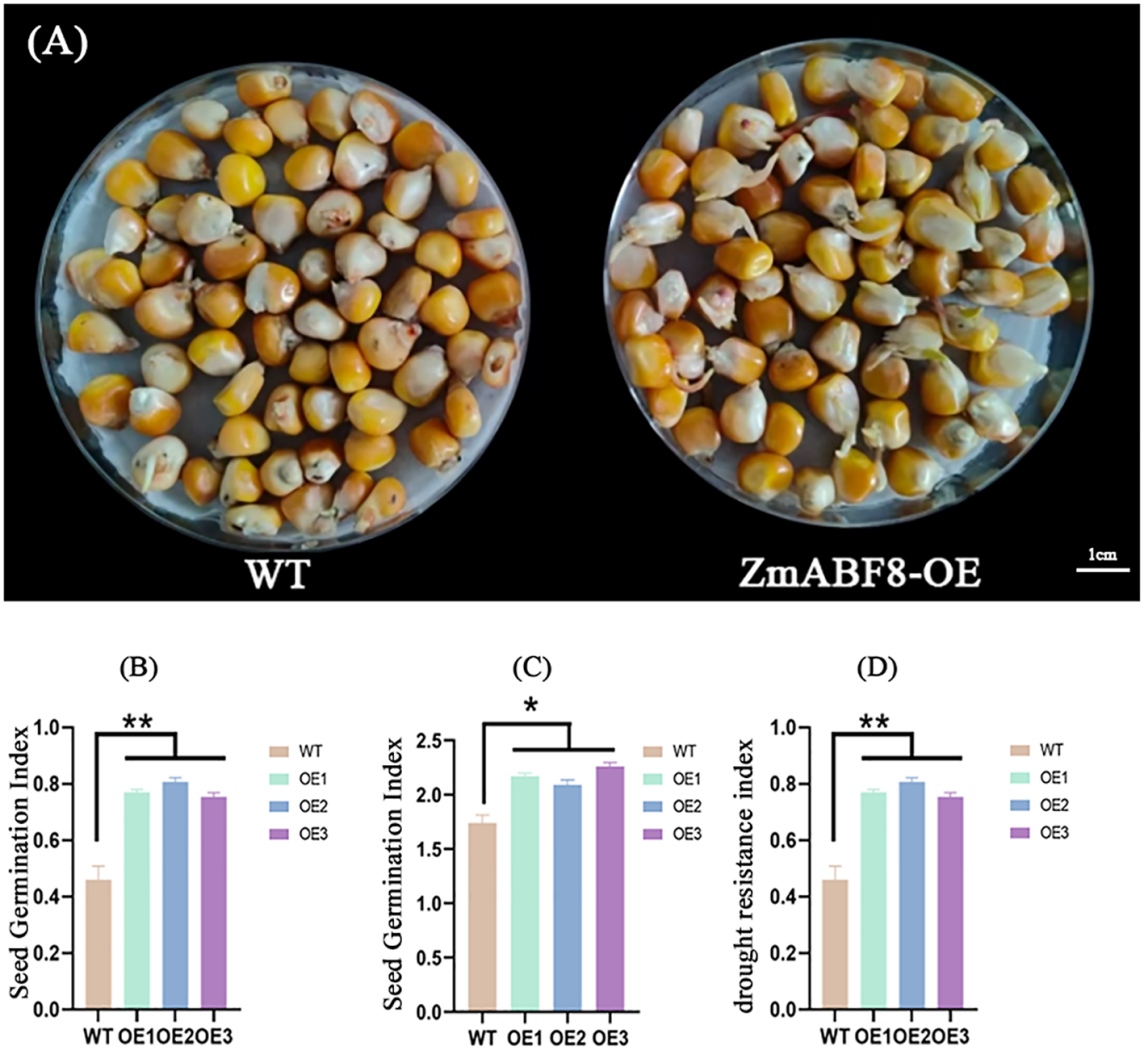
Phenotype analysis of seed germination in *ZmABF8* overexpressing plants under drought stress. **(A)** *ZmABF8* positive plants and wild-type seed germination phenotypes under drought stress. **(B)** Analysis of Seed Germination Index between *ZmABF8* Positive Plants and Wild Type under Drought Stress. **(C)** Analysis of Seed Germination Index between ZmABF8 Positive Plants and Wild Type under Normal Conditions. **(D)** Comparative analysis of drought resistance index between *ZmABF8* positive plants and wild-type plants. Data are shown as mean ± SD from three independent experiments. OE1-OE3 are three transformation events of ZmABF8 positive plants.

**Figure 12:**
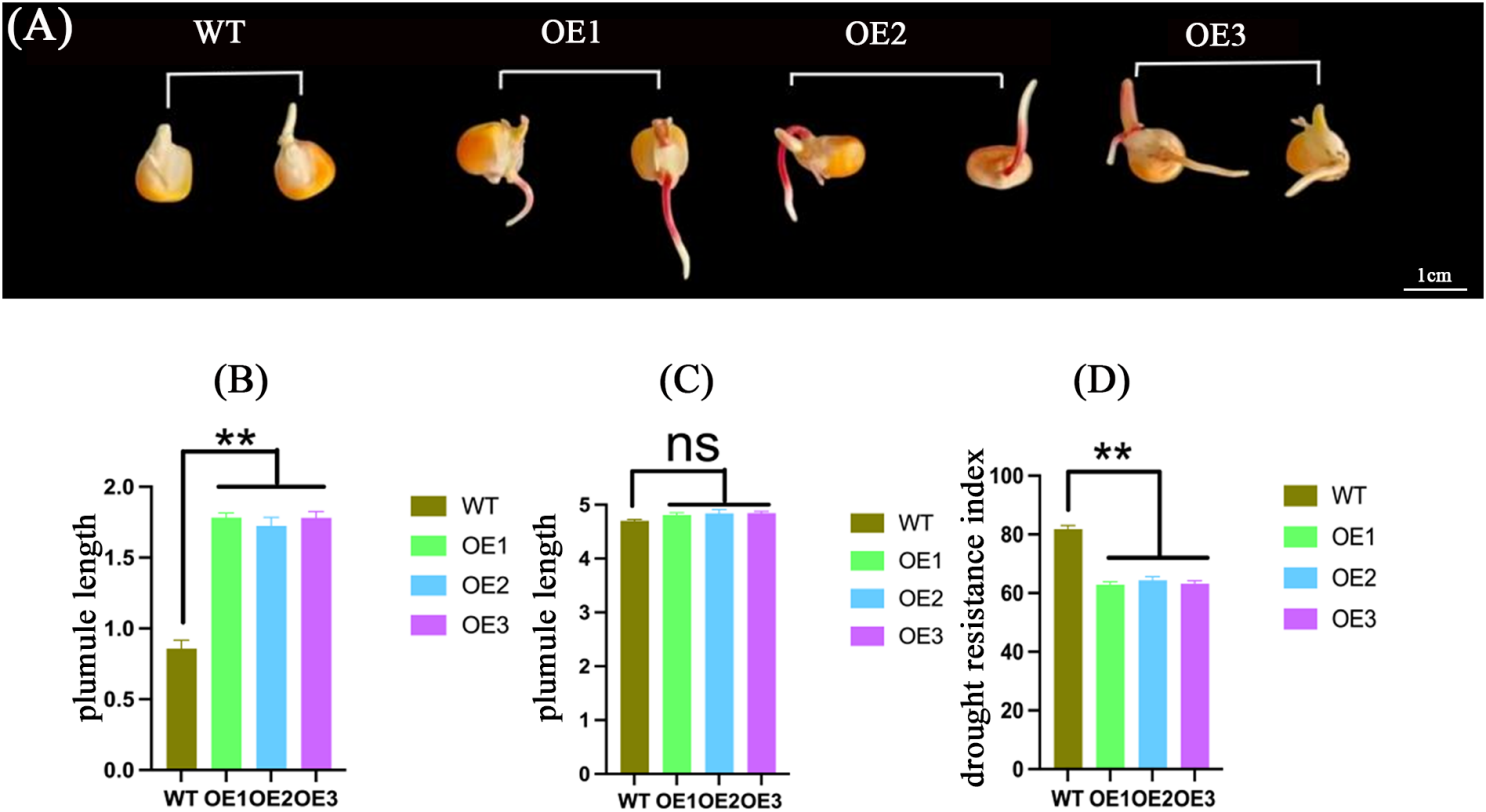
Phenotype analysis of seed plumule length and plumule damage rate in *ZmABF8* overexpressing plants under drought stress. **(A)** Phenotype of plumule development in *ZmABF8* positive plants and wild-type seeds under drought stress. **(B)** Analysis of plumule length of *ZmABF8* positive plants and wild-type seeds under drought stress. **(C)** Analysis of plumule length between *ZmABF8* positive plants and wild-type seeds under normal conditions. **(D)** Comparative analysis of drought resistance index between *ZmABF8* positive plants and wild-type plants. Data are shown as mean ± SD from three independent experiments. OE1-OE3 are three transformation events of *ZmABF8* positive plants.

**Figure 13:**
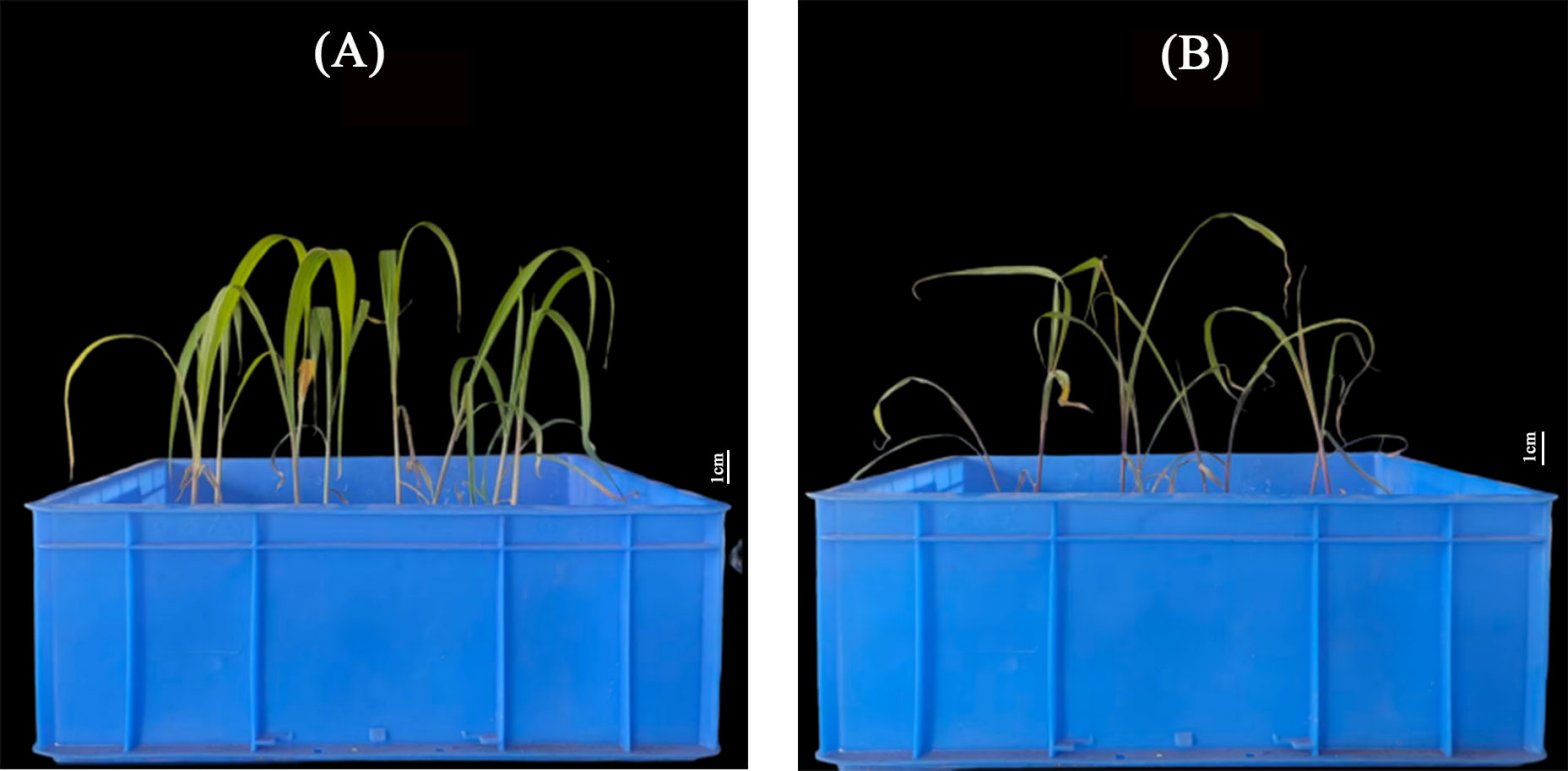
Phenotype of *ZmABF8* overexpressing plants at the seedling stage under drought stress. **(A)** *ZmABF8*-OE after 7 days of drought stress during the three-leaf stage of maize; **(B)** *ZmABF8*-WT after 7 days of drought stress during the three-leaf stage of maize;.

Based on the representativeness of *ZmABF8* in abiotic stress, especially drought stress, demonstrated in the above research results, this study cloned and created *ZmABF8* overexpressing positive plants. the seed germination index, drought resistance index, as well as the length of the seedling shoot and the rate of seedling damage in a high osmotic environment can intuitively reflect the drought tolerance of maize. This study analyzes the germination index and drought resistance index of *ZmABF8* positive plants, indicating that under drought stress, The seed germination phenotype of ZmABF8 overexpressing plants under drought stress is shown in **Figure 11A**. The germination index and drought resistance index of the transgenic plant seeds were significantly higher than those of the control (**Figures 11B, D**). Research on the embryo length and seed embryo injury rate shows that under drought stress, the embryo length of transgenic plants ranges from 1.73 to 1.82 cm, which is significantly longer than that of the control (**Figure 12B**). The germination injury rate of transgenic plants is significantly lower than that of the control group (**Figure 12**). Additionally, this study evaluated the drought-resistant phenotype of *ZmABF8* positive plants during the seedling stage. The results indicate that after 7 days of drought stress during the three-leaf stage of maize, the transgenic plants showed no significant signs of wilting, while the control plants exhibited significant yellowing and wilting (**Figures 13A, B**). The above experiments demonstrate that *ZmABF8* positively regulates drought resistance in maize, significantly enhancing the drought tolerance of transgenic plants.

**Figure 14:**
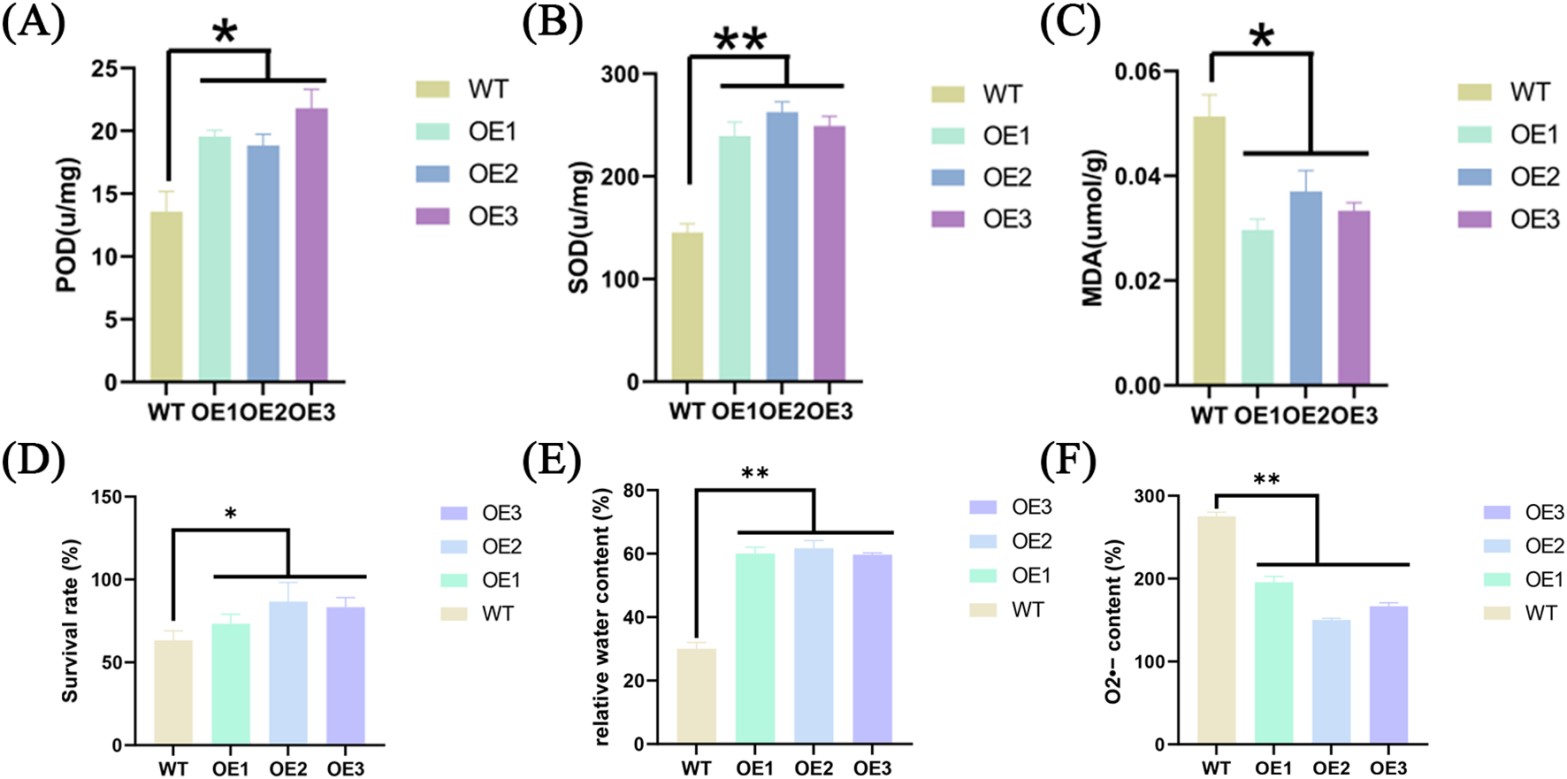
Physiological and biochemical index detection of *ZmABF8* overexpressing plants. **(A)** Peroxidase (POD) activity; **(B)** superoxide dismutase (SOD) activity; **(C)** malondialdehyde (MDA) content; **(D)** survival rate; **(E)** relative water content; **(F)** O_2_^-^ content.Data are presented as mean ± SD from three independent experiments (n = 3). OE1–OE3 represent three independent transgenic lines of *ZmABF*8 overexpression plants. Statistical differences between each OE line and WT were analyzed using Student’s t-test. Asterisks indicate significant differences compared with WT (*P < 0.05, **P < 0.01), and ns indicates no significant difference.

After experiencing drought stress, the increase in reactive oxygen species (ROS) can cause persistent damage to plant cells. Therefore, a plant’s ability to suppress ROS levels is an important indicator of its drought tolerance. This study investigated the activities of POD, SOD, and MDA in *ZmABF8* positive plants under drought stress conditions. Higher activities of POD and SOD indicate a stronger ability of theplants to scavenge reactive oxygen species, while lower levels of MDA suggest reduced cellular damage from ROS (**Figures 14A–C**). In addition, the overexpressing plants exhibited higher survival rates and relative water content, while O2− content was significantly reduced under drought stress (**Figures 14D–F**). The research results indicate that the POD enzyme activity in *ZmABF8* positive plants is significantly higher than that of the control plants, while the SOD enzyme activity reached a highly significant level compared to the control. Additionally, the MDA content is significantly lower than that of the control. This suggests that *ZmABF8* positive plants can actively respond to drought stress by enhancing the levels of POD and SOD, thereby mitigating the damage caused by reactive oxygen species to plant cells.

## 3 Discussion

*ZmABF* belongs to a subgroup of the basic leucine zipper (bZIP) transcription factor family (Bensmihen, 2005). Numerous studies have confirmed the importance of ABA signal transduction in plant stress responses (Waadt et al., 2022). The ABF family has been confirmed to play an indispensable role in the ABA signaling pathway for plant stress adaptation (Fujita et al., 2011). Currently, research on the *ABF* family in maize remains unclear. A total of nine ABF family members were identified in Arabidopsis (Yoshida et al., 2010). Based on the similarity to Arabidopsis ABF family proteins and the comparison of conserved domains, we identified 18 ABF family members in maize. In this study, an evolutionary tree for the gene family was constructed, along with analyses of conserved domains, chromosomal localization, synteny, and expression levels. The genes of this family are distributed across chromosomes 1 to 9 of maize, and there is only one bZIP conserved domain. However, in terms of motif distribution, there is a diversity within the family. *ZmABF* is classified into four phylogenetic branches (Group I, II, IV and V), with one branch (Group III) less than that in Arabidopsis and sorghum, suggesting that this sub-group might have undergone loss or functional replacement during the evolution of maize.As an important subfamily of transcription factors in plants, the subcellular localization prediction of *ZmABF* is consistently found in the nucleus, and the subcellular localization results of *ZmABF8* also confirm the accuracy of these predictions. In the analysis of cis-acting elements in the promoter regions of the *ZmABF* gene family, all members of the family contain cis-acting elements such as ARE and ABRE, which are closely related to ABA signal transduction. The promoter region of the *ZmABF5* gene contains up to 9 ABREs and 4 AREs, making it the gene with the highest number of ABA-related cis-acting elements among the family members. This indicates that *ZmABF* responds very actively and vigorously to ABA signals (Fujita et al., 2011).

The *ABF* gene family acts as a multimodal stress sensor, dynamically activating the ABA signaling cascade to coordinate downstream stress response networks (Zhang et al., 2025). In the analysis of interaction proteins within the ABF family of maize, a total of 20 interacting genes were predicted, among which 10 proteins exhibited a compatibility score of ≥0.8. Notably, all 10 proteins belong to the same kinase family, namely the Serine/Threonine protein kinase SnRK2. This indicates that this kinase family is likely a key factor in regulating the activity, stability, and function of ABF proteins. Research indicates that the SnRK2 gene family is a core regulatory factor in the ABA signaling pathway (Hirayama and Umezawa, 2010). SnRK2 is activated in the presence of ABA, enhancing the transcriptional activity of ABF through the phosphorylation of ABF, which promotes the accumulation of ABF protein and enhances its ability to bind to downstream DNA (Fujita et al., 2013). This gene family regulates plant adaptation to drought through three physiological mechanisms: stomatal closure regulation, osmotic pressure regulation, and activation of reactive oxygen species scavenging systems (Zhang et al., 2025). The ABF gene family has been confirmed to play a significant role in the abiotic stress adaptability of rice, wheat, potato, cotton (Rikiishi et al., 2010; Miyazono et al., 2012; Kerr et al., 2018; Liu et al., 2019.) Analysis of the expression patterns of the maize ABF gene family indicates that the primary expression sites of this gene family are the roots and leaves. This is consistent with the role of closing stomata to reduce water loss and the function of root osmotic pressure in promoting the absorption of soil moisture. In wheat, TaABF17-19 (ABF17-19) is significantly upregulated under drought stress (Yang et al., 2024). In tomatoes, 10 ABI5 family genes are significantly upregulated under ABA induction (Pan et al., 2023); in apples, ABF3 (ABF3) is significantly upregulated under drought stress, and overexpression of this gene significantly enhances drought tolerance (Rui et al., 2022). In maize, the expression levels of *ZmABF3*, *ZmABF5*, *ZmABF7*, *ZmABF8*, and *ZmABF12* are significantly upregulated under heat stress, cold stress, and drought stress. Moreover, we conducted relative expression validation for the aforementioned five genes, and the results are consistent with the RNA-seq data. In rice, *OsABF1* (ABF1) enhances drought tolerance by regulating the expression of genes such as COR413-TM1 (Zhang et al., 2017). In this study, the ABF gene family in maize is found to be completely collinear with that in rice, further supporting the role of ABF in maize’s response to drought.

To further validate the role of the ABF family in drought stress, this study cloned the *ZmABF8* gene, which is most sensitive to drought response, and successfully created positive overexpression plants using the maize inbred line B73 as the recipient through Agrobacterium-mediated transformation. Phenotypic analysis indicated that, under drought stress conditions, the seed germination, drought resistance index, and embryo length were all enhanced compared to the wild type, while the embryo injury rate was significantly reduced, and drought tolerance during the seedling stage was markedly improved. Studies on reactive oxygen species physiological indicators showed that the overexpression of the *ZmABF8* gene significantly increased the activity of superoxide dismutase and peroxidase, and significantly reduced cell damage caused by reactive oxygen species under drought stress. Previous studies on model plants have confirmed that the ABF gene is highly dependent on the ABA signaling pathway in response to abiotic stress (Li et al., 2022). The response of *ZmABF8* to drought is likely derived from the activation of *SnRK2* by ABA, which enhances its transcriptional activity and stimulates the reactive oxygen species (ROS) scavenging system, thereby mitigating cellular damage and improving drought resistance in maize. Although these findings lay a solid theoretical foundation for elucidating the drought resistance mechanism of *ZmABF8*, the drought response mechanism mediated by *ZmABF8* is quite complex. Further research is needed to determine whether *ZmABF8* achieves functional integration of these signals in maize.

## 4 Materials and methods

### 4.1 Identification of *ZmABF* gene family in maize, analysis of protein physicochemical properties, and prediction of subcellular localization

The genomic and protein sequence data of maize are sourced from the Maize -GDB database (https://maizegdb.org/, accessed August 19, 2025), with Zm-B73-REFERENCE-NAM-4.0 selected as the reference sequence version. The protein sequence of ABF from *Arabidopsis thaliana* was obtained from the TAIR database (https://www.arabidopsis.org/, accessed August 19, 2025). The ABF gene family was identified across the whole genome using a combination of BLASTP and Hidden Markov Model (HMM) search methods. Using the Arabidopsis ABF protein sequence as a template, a local BLASTP search of the maize proteome was conducted using the ‘Blast Compare Two Seqs’ module of TBtools software (v2.101) (E-value ≤ 1×10^-5^). Concurrently, the HMM model of the bZIP domain (PF00170) was obtained from the Pfam database (http://pfam.xfam.org/, accessed August 19, 2025), and filtered using the ‘Simple HMM Search’ function of TBtools. The intersection of the results from both searches was taken as candidate genes. Finally, the candidate proteins were verified for the presence of complete bZIP conserved domains using the NCBI Conserved Domain Database (CDD, https://www.ncbi.nlm.nih.gov/Structure/bwrpsb/bwrpsb.cgi, accessed August 20, 2025) and the SMART online tool (https://smart.embl.de/, accessed August 20, 2025), which helped to identify members of the maize ABF gene family. Based on their physical locations on the chromosomes, they were designated as *ZmABF1* to *ZmABF18*. The visualization of chromosome localization was accomplished using TBtools v. 2.142 software.

The physicochemical properties of proteins were analyzed using the Expasy online tool ProtParam (https://web.expasy.org/protparam/, accessed August 21, 2025), which provides information on the number of amino acids, molecular weight, and theoretical isoelectric point (pI) of the proteins encoded by each member. The subcellular localization of the proteins was predicted using the WoLF PSORT online tool (https://wolfpsort.hgc.jp/, accessed August 21, 2025).

### 4.2 Systematic evolution, gene structure, and conserved motif analysis of maize ABF gene family

The construction of the phylogenetic tree utilized ClustalW to perform a multiple sequence alignment of the ABF protein sequences from maize, *Arabidopsis thaliana*, and millet (*Setaria italica*) (Sievers et al., 2011). The phylogenetic tree was constructed using the Maximum Likelihood (ML) method implemented in MEGA11 software, with a Bootstrap value set to 1000 repetitions to assess branch reliability (Kumar et al., 2018). The tree was visualized using the iTOL online platform (https://itol.embl.de, accessed August 25, 2025). Conservative motif analysis was conducted using the online MEME Suite 5.5.7 tool (https://meme-suite.org/meme/tools/meme, accessed August 25, 2025) to identify protein conservative motifs. The parameters were set to a maximum of 20 motifs with a width range of 6 to 50 amino acids. Gene structure analysis was based on the maize genome annotation file, utilizing the ‘Visualize Gene Structure (GFF3)’ function of TBtools to generate gene structure diagrams, illustrating information on exons, introns, and untranslated regions (Chen et al., 2020).

### 4.3 Analysis of collinearity, protein-protein interaction, cis acting elements and miRNA targets of maize ABF gene family

We generated a protein-protein interaction (PPI) network via the STRING database (https://string-db.org/, accessed August 29, 2025), applying an interaction score threshold of ≥0.4 and restricting the false discovery rate (FDR) to 5%. The analysis of cis-acting elements for the *ZmABF* gene was initiated by extracting the promoter sequences located 2000 bp upstream of the transcription start sites (TSS) of each *ZmABF* gene using TBtools. The sequences were submitted to the PlantCARE database (https://bioinformatics.psb.ugent.be/webtools/plantcare/html/, accessed August 28, 2025) for the prediction of cis-regulatory elements. Additionally, the psRNATarget online tool (https://www.zhaolab.org/psRNATarget/, accessed August 28, 2025) was utilized with default parameters to predict miRNAs that may target the mRNA of the *ZmABF* gene (Melnikova et al., 2016).

### 4.4 Subcellular localization of *ZmABF8*

In this study, the most actively drought-responsive gene *ABF8* from the ABF family was cloned. the roots of the B73 maize inbred line were washed and then immersed in a 25% PEG 6000 aqueous solution for 2 hours. Extract total RNA from stressed leaves of maize and reverse transcribe it into cDNA. The cloning vector pMD-18T was used, and a seamless cloning kit was used as a template to construct the transient expression vector pCAMBIA1302-*ZmABF8*-GFP based on homologous recombination principle. The enzyme cleavage sites of the vector were BglII and NcoI. The instantaneous transformation and subcellular localization procedures of *Nicotiana benthamiana* are detailed in the study by Jianbo Fei (Liu et al., 2024b).

### 4.5 RNA extraction and qRT PCR

Transcriptome data were downloaded from the Maize Public Expression Database qTeller (https://qteller.maizegdb.org, accessed September 2, 2025), encompassing RNA-seq datasets from various tissues and non-biotic stress treatments. Among them, the drought stress treatment involved subjecting three-leaf-stage corn seedlings to a 7-day natural drought (ceasing watering). The drought stress samples (wt_Drought_T0 and wt_Drought_T7) in the public expression data originated from the studies of Stelpflug et al., Forestan et al., and Waters et al (Forestan et al., 2016; Stelpflug et al., 2016; Waters et al., 2017). The processing of samples for qRT-PCR validation: The root systems of B73 maize inbred line three-leaf stage seedlings were washed and then placed in a 25% (w/v) PEG 6000 aqueous solution to simulate drought stress. After 2 hours of treatment, the leaves were collected to extract total RNA. The control plants were treated in distilled water during the same period. Cold stress treatment was carried out in a 4 °C incubator for 6 hours, and heat stress treatment was conducted in a 42 °C incubator for 6 hours. Three biological replicates were set for each treatment.The expression levels (FPKM values) of *ZmABF* family members in various samples were extracted and a heatmap illustrating tissue specificity and stress response expression was generated using the pheatmap package in R (v4.2.2). Total RNA from different maize tissues and stress-treated samples was extracted using the TRIzol method, and cDNA was synthesized through reverse transcription using the HiScript II 1st Strand cDNA Synthesis Kit. qRT-PCR reactions were conducted using SYBR Green Master Mix on the LightCycler^®^ 480 II real-time fluorescence quantitative PCR system. Each sample was set up with three biological replicates and three technical replicates. The relative expression levels of the genes were calculated using the 2^-^ΔΔCt method, with maize Actin or Ubiquitin genes serving as internal references. Perform a Student t-test using SPSS Statistics 27 software, with a significance level set at *p* < 0.05 (Chicco et al., 2025).

### 4.6 Creation and functional validation of *ZmABF8* overexpression material

The overexpression vector was constructed using the cDNA of the maize inbred line B73 as a template to clone the complete coding sequence (CDS) of the *ZmABF8* gene. This sequence was then inserted into the modified plant overexpression vector pCAMBIA3301, which contains the Ubi promoter, resulting in the recombinant plasmid pUbi::*ZmABF8*. The recombinant vector was introduced into the maize inbred line B73 via Agrobacterium-mediated transformation of immature embryos (Sidorov and Duncan, 2009). T0 generation resistant plants were obtained through herbicide screening and regeneration culture, and stable high-expressing homozygous T2 lines of *ZmABF8* were selected for subsequent experiments through PCR screening.

To assess the oxidative stress levels and drought resistance of plants, key physiological indicators such as superoxide dismutase (SOD), peroxidase (POD), and malondialdehyde (MDA) were measured during the seedling stage (Li et al., 2024). Calculation of Seed Germination Index and Drought Resistance Index of Germination. The calculation method of the seed germination index (PI) is as follows:


PI = 1.00 nd2+ 0.75 nd4+ 0.50 nd6+ 0.25 nd8

$$PI_{ck}=\;1.00\;nd2_{ck}+\;0.75\;nd4_{ck}+\;0.50\;nd6_{ck}+\;0.25\;nd8_{ck}$$

$$PI_{s}=\;1.00\;nd2_{s}+\;0.75\;nd4_{s}+\;0.50\;nd6_{s}+\;0.25\;s$$

Among them, nd_2_, nd_4_, nd_6_ and nd_8_ represent the germination rates on the 2nd, 4th, 6th and 8th days respectively.

The calculation formula for the Germination Drought Resistance Index (GDRI) is:


$$GDRI=\frac{PI_{s}}{PI_{ck}}\times 100\%$$

Among them, $PI_{s}$ and $PI_{ck}$ respectively represent the available index under drought stress conditions and control conditions.The activity of superoxide dismutase (SOD) was determined using the nitroblue tetrazolium (NBT) photoreduction method (Heath and Packer, 1968). The activity of peroxidase (POD) was measured using the guaiacol method (Beauchamp and Fridovich, 1971). The content of malondialdehyde (MDA) was quantified using the thiobarbituric acid (TBA) method (Draper et al., 1993), which indicates the extent of membrane lipid peroxidation. All physiological indicators were measured with three biological replicates. Data were statistically analyzed using SPSS Statistics 27, and the chart data are presented as mean ± standard deviation.

The determination of relative water content (RWC) was conducted following the method described in previous studies (Gaxiola et al., 2001). RWC was calculated using the formula:


$$RWC=[\frac{FW-DW}{TW-DW}]\times 100\%$$

Uniformly grown three-leaf stage *ZmABF8*-OE and wild type (WT) were subjected to drought stress. The treatment lasted for a specific period of 7 days until obvious dehydration signs appeared in the WT seedlings. Calculation: After 3 to 5 days of recovery, seedlings with leaves re-expanded and green tissue maintained were regarded as survivors (Wang et al., 2021). The survival rate was calculated using the following formula:


$$Survival\;Rate(\%)=\frac{Number\;of\;surviving\;seedlings}{Total\;number\;of\;seedlings\;treated}\times 100\%$$

Superoxide anion (O_2_^-^) content was determined using the nitroblue tetrazolium (NBT) reduction method. Fresh plant tissues (approximately 0.3 g) were homogenized in 50 mM phosphate buffer (pH 7.8) under ice-cold conditions and centrifuged at 12, 000 × g for 15 min at 4 °C. The reaction mixture contained the supernatant, 50 mM phosphate buffer (pH 7.8), and 0.1% (w/v) NBT. After incubation at 25 °C for 20 min in the dark, the absorbance was measured at 560 nm. The O_2_^-^ level was expressed based on the absorbance value (Jabs, 1999).

### 4.7 Stress treatments for qRT-PCR analysis

Maize seedlings at the three-leaf stage were subjected to distinct abiotic stress treatments. For drought stress, seedlings were treated with a 25% PEG-6000 solution, and leaf samples were collected at 0 days (0T) and 7 days (7T) post-treatment. For cold stress, seedlings were incubated at 4 °C for 24 h. For heat stress, seedlings were exposed to 42 °C for 24 h. Leaf samples were harvested immediately after each treatment, rapidly frozen in liquid nitrogen, and stored at −80 °C until RNA extraction. Each treatment comprised three independent biological replicates.

## 5 Conclusions

This study presents the first comprehensive genomic characterization of the ABF gene family in maize, revealing its evolutionary dynamics and developmental regulatory patterns. In summary, 18 ABF genes have been identified in maize. Through systematic bioinformatics analysis, the ABF gene family has been classified into four subgroups, all of which are expressed in the nucleus. The promoter regions contain a high proportion of ABA-related cis-elements, with a maximum of 13 present. Each gene in the family contains a bZIP conserved domain, but the motifs included are quite diverse, indicating that there is no redundancy in function. Expression analysis reveals that ABF genes are primarily expressed in the roots and leaves, and are induced by abiotic stress, particularly responding rapidly to drought stress. Overexpression of *ZmABF8* in maize can significantly enhance seed germination rates and drought resistance during the seedling stage under drought stress. It markedly increases the activities of POD and SOD in maize, thereby reducing cellular damage.

## Data Availability

The original contributions presented in the study are included in the article/**Supplementary Material**. Further inquiries can be directed to the corresponding authors.
