# Supplementary material for: Genome-wide identification and functional characterization of the ABF gene family in maize reveals ZmABF8 as a key regulator of drought tolerance
Source: Front Plant Sci. 2026 May 21;17:1849922. doi: 10.3389/fpls.2026.1849922 (PMC13233282; doi:10.3389/fpls.2026.1849922)
Supplement: Supplementary file 1 [file SupplementaryFile1.docx]

Supplementary Material

# Supplementary Tables

**TableS1. The protein characteristics of ZmABF family members in maize.**

| **Sequence ID** | **gene name** | **Number of Amino Acid** | **MW(kDa)** | **pI** | **Instability Index** | **Aliphatic Index** | **Grand Average of Hydropathicity** | **Predicted Location(s)** |
| --- | --- | --- | --- | --- | --- | --- | --- | --- |
| Zm00001d031790 | ZmABF1 | 350 | 36938.67 | 6.52 | 49.91 | 67.86 | -0.42 | nucl |
| Zm00001d005884 | ZmABF2 | 295 | 32164.68 | 9.43 | 57.08 | 66.58 | -0.574 | nucl |
| Zm00001d042721 | ZmABF3 | 260 | 27553.04 | 8.95 | 74.98 | 65.38 | -0.673 | nucl |
| Zm00001d042779 | ZmABF4 | 383 | 40464.4 | 7.66 | 57.35 | 63.24 | -0.508 | nucl |
| Zm00001d043117 | ZmABF5 | 333 | 36247.53 | 6.69 | 59.57 | 72.97 | -0.71 | nucl |
| Zm00001d050018 | ZmABF6 | 356 | 37692.54 | 5.44 | 49.79 | 69.97 | -0.379 | nucl |
| Zm00001d018178 | ZmABF7 | 351 | 37851.36 | 9.56 | 67.18 | 71.45 | -0.459 | nucl |
| Zm00001d018571 | ZmABF8 | 171 | 18616.89 | 9.22 | 83.06 | 74.21 | -0.743 | nucl |
| Zm00001d036392 | ZmABF9 | 161 | 17874.22 | 9.26 | 56.88 | 78.88 | -0.472 | nucl |
| Zm00001d037170 | ZmABF10 | 338 | 36351.31 | 8.59 | 39.72 | 77.4 | -0.401 | nucl |
| Zm00001d038311 | ZmABF11 | 254 | 27087.44 | 7.76 | 68.69 | 64.33 | -0.585 | nucl |
| Zm00001d020711 | ZmABF12 | 405 | 43145.39 | 5.58 | 49.42 | 64.44 | -0.452 | nucl |
| Zm00001d022550 | ZmABF13 | 285 | 31162.42 | 9.98 | 73.85 | 66.49 | -0.851 | nucl |
| Zm00001d022613 | ZmABF14 | 204 | 21507.99 | 9.16 | 57.66 | 66.62 | -0.421 | nucl |
| Zm00001d010638 | ZmABF15 | 344 | 37498.03 | 6.05 | 52.87 | 75.7 | -0.574 | nucl |
| Zm00001d012273 | ZmABF16 | 244 | 25469.56 | 6.05 | 65.44 | 65.7 | -0.493 | nucl |
| Zm00001d012296 | ZmABF17 | 412 | 43446.87 | 5.99 | 58.51 | 58.37 | -0.514 | nucl |
| Zm00001d044940 | ZmABF18 | 351 | 37568.66 | 7.84 | 53.83 | 75.44 | -0.418 | nucl |

**Table S2.MiRNA prediction of ZmABF gene**

| **miRNA_Acc.** | **Target_Acc.** | **Expectation** | **miRNA_start** | **miRNA_end** | **Target_start** | **Target_end** | **Inhibition** | **Multiplicity** |
| --- | --- | --- | --- | --- | --- | --- | --- | --- |
| zma-miR171f-5p | Zm00001d010638 | 5 | 1 | 21 | 894 | 916 | Cleavage | 1 |
| zma-miR172b-5p | Zm00001d010638 | 5 | 1 | 20 | 186 | 205 | Translation | 1 |
| zma-miR172d-5p | Zm00001d010638 | 5 | 1 | 20 | 186 | 205 | Translation | 1 |
| zma-miR160f-3p | Zm00001d012273 | 4.5 | 1 | 21 | 43 | 63 | Cleavage | 1 |
| zma-miR156e-3p | Zm00001d012273 | 5 | 1 | 22 | 262 | 283 | Translation | 2 |
| zma-miR156e-3p | Zm00001d012273 | 5 | 1 | 22 | 85 | 106 | Cleavage | 2 |
| zma-miR396g-3p | Zm00001d012273 | 5 | 1 | 21 | 680 | 700 | Cleavage | 1 |
| zma-miR159e-5p | Zm00001d012296 | 4 | 1 | 21 | 151 | 171 | Cleavage | 1 |
| zma-miR1432-3p | Zm00001d012296 | 5 | 1 | 22 | 546 | 567 | Cleavage | 1 |
| zma-miR166a-3p | Zm00001d012296 | 5 | 1 | 21 | 420 | 440 | Cleavage | 1 |
| zma-miR166b-3p | Zm00001d012296 | 5 | 1 | 20 | 421 | 440 | Cleavage | 1 |
| zma-miR166c-3p | Zm00001d012296 | 5 | 1 | 20 | 421 | 440 | Cleavage | 1 |
| zma-miR166d-3p | Zm00001d012296 | 5 | 1 | 20 | 421 | 440 | Cleavage | 1 |
| zma-miR166e | Zm00001d012296 | 5 | 1 | 20 | 421 | 440 | Cleavage | 1 |
| zma-miR166f | Zm00001d012296 | 5 | 1 | 20 | 421 | 440 | Cleavage | 1 |

**Continued tableS2.MiRNA prediction of ZmABF gene**

| **miRNA_Acc.** | | **Target_Acc.** | **Expectation** | | **miRNA_start** | | **miRNA_end** | | **Target_start** | | **Target_end** | | **Inhibition** | | **Multiplicity** | |
| --- | --- | --- | --- | --- | --- | --- | --- | --- | --- | --- | --- | --- | --- | --- | --- | --- |
| zma-miR166g-3p | Zm00001d012296 | | | 5 | | 1 | | 20 | | 421 | | 440 | | Cleavage | | 1 |
| zma-miR166h-3p | Zm00001d012296 | | | 5 | | 1 | | 20 | | 421 | | 440 | | Cleavage | | 1 |
| zma-miR166i-3p | Zm00001d012296 | | | 5 | | 1 | | 20 | | 421 | | 440 | | Cleavage | | 1 |
| zma-miR166l-3p | Zm00001d012296 | | | 5 | | 1 | | 21 | | 420 | | 440 | | Cleavage | | 1 |
| zma-miR166m-3p | Zm00001d012296 | | | 5 | | 1 | | 21 | | 420 | | 440 | | Cleavage | | 1 |
| zma-miR399f-5p | Zm00001d012296 | | | 5 | | 1 | | 22 | | 892 | | 913 | | Cleavage | | 1 |
| zma-miR395a-5p | Zm00001d018178 | | | 4.5 | | 1 | | 23 | | 757 | | 779 | | Cleavage | | 1 |
| zma-miR167d-3p | Zm00001d018178 | | | 5 | | 1 | | 23 | | 24 | | 46 | | Cleavage | | 1 |
| zma-miR2275a-3p | Zm00001d018178 | | | 5 | | 1 | | 22 | | 699 | | 720 | | Cleavage | | 1 |
| zma-miR397a-5p | Zm00001d018571 | | | 5 | | 1 | | 21 | | 317 | | 337 | | Cleavage | | 1 |
| zma-miR397b-5p | Zm00001d018571 | | | 5 | | 1 | | 21 | | 317 | | 337 | | Cleavage | | 1 |
| zma-miR529-3p | Zm00001d020711 | | | 5 | | 1 | | 21 | | 796 | | 816 | | Translation | | 1 |
| zma-miR164a-3p | Zm00001d022550 | | | 3.5 | | 1 | | 21 | | 645 | | 665 | | Cleavage | | 1 |
| zma-miR2275a-3p | Zm00001d022550 | | | 4.5 | | 1 | | 22 | | 642 | | 663 | | Cleavage | | 1 |
| zma-miR1432-3p | Zm00001d022550 | | | 5 | | 1 | | 22 | | 285 | | 306 | | Cleavage | | 1 |

**Continued tableS2.MiRNA prediction of ZmABF gene**

| **miRNA_Acc.** | | **Target_Acc.** | **Expectation** | | **miRNA_start** | | **miRNA_end** | | **Target_start** | | **Target_end** | | **Inhibition** | | **Multiplicity** | |
| --- | --- | --- | --- | --- | --- | --- | --- | --- | --- | --- | --- | --- | --- | --- | --- | --- |
| zma-miR2275d-3p | Zm00001d022550 | | | 5 | | 1 | | 22 | | 642 | | 663 | | Cleavage | | 1 |
| zma-miR169f-3p | Zm00001d022613 | | | 4.5 | | 1 | | 21 | | 333 | | 353 | | Cleavage | | 1 |
| zma-miR167c-3p | Zm00001d022613 | | | 5 | | 1 | | 23 | | 317 | | 339 | | Cleavage | | 1 |
| zma-miR169r-3p | Zm00001d022613 | | | 5 | | 1 | | 21 | | 333 | | 353 | | Cleavage | | 1 |
| zma-miR399d-5p | Zm00001d031790 | | | 4 | | 1 | | 21 | | 177 | | 197 | | Cleavage | | 1 |
| zma-miR156a-3p | Zm00001d031790 | | | 4.5 | | 1 | | 22 | | 10 | | 31 | | Cleavage | | 1 |
| zma-miR156d-3p | Zm00001d031790 | | | 5 | | 1 | | 22 | | 10 | | 31 | | Cleavage | | 1 |
| zma-miR156f-3p | Zm00001d031790 | | | 5 | | 1 | | 22 | | 10 | | 31 | | Cleavage | | 1 |
| zma-miR156g-3p | Zm00001d031790 | | | 5 | | 1 | | 22 | | 10 | | 31 | | Cleavage | | 1 |
| zma-miR156k-3p | Zm00001d031790 | | | 5 | | 1 | | 22 | | 10 | | 31 | | Cleavage | | 1 |
| zma-miR529-3p | Zm00001d031790 | | | 5 | | 1 | | 21 | | 834 | | 854 | | Cleavage | | 1 |
| zma-miR2118a | Zm00001d038311 | | | 5 | | 1 | | 22 | | 424 | | 445 | | Translation | | 1 |
| zma-miR160d-3p | Zm00001d042721 | | | 4.5 | | 1 | | 21 | | 102 | | 122 | | Cleavage | | 1 |
| zma-miR156e-3p | Zm00001d042721 | | | 5 | | 1 | | 22 | | 262 | | 283 | | Translation | | 2 |
| zma-miR156e-3p | Zm00001d042721 | | | 5 | | 1 | | 22 | | 85 | | 106 | | Cleavage | | 2 |

**Continued tableS2.MiRNA prediction of ZmABF gene**

| **miRNA_Acc.** | | **Target_Acc.** | **Expectation** | | **miRNA_start** | | **miRNA_end** | | **Target_start** | | **Target_end** | | **Inhibition** | | **Multiplicity** | |
| --- | --- | --- | --- | --- | --- | --- | --- | --- | --- | --- | --- | --- | --- | --- | --- | --- |
| zma-miR168a-3p | Zm00001d043117 | | | 5 | | 1 | | 20 | | 425 | | 444 | | Cleavage | | 1 |
| zma-miR528a-3p | Zm00001d043117 | | | 5 | | 1 | | 21 | | 37 | | 57 | | Cleavage | | 1 |
| zma-miR528b-3p | Zm00001d043117 | | | 5 | | 1 | | 21 | | 37 | | 57 | | Cleavage | | 1 |
| zma-miR167e-3p | Zm00001d044940 | | | 4 | | 1 | | 22 | | 810 | | 831 | | Cleavage | | 1 |
| zma-miR167f-3p | Zm00001d044940 | | | 4 | | 1 | | 22 | | 810 | | 831 | | Cleavage | | 1 |
| zma-miR396a-5p | Zm00001d050018 | | | 5 | | 1 | | 21 | | 811 | | 831 | | Cleavage | | 1 |
| zma-miR396b-5p | Zm00001d050018 | | | 5 | | 1 | | 21 | | 811 | | 831 | | Cleavage | | 1 |
| zma-miR396e-5p | Zm00001d050018 | | | 5 | | 1 | | 21 | | 811 | | 831 | | Cleavage | | 1 |
| zma-miR396f-5p | Zm00001d050018 | | | 5 | | 1 | | 21 | | 811 | | 831 | | Cleavage | | 1 |
| zma-miR529-3p | Zm00001d050018 | | | 5 | | 1 | | 21 | | 852 | | 872 | | Cleavage | | 1 |

**Table S3. Primers for ZmABF gene in qRT PCR experiment**

| **Gene name** | **Forward primer** | **Reserve primer Reserve primer** |
| --- | --- | --- |
| **ZmABF8(Zm00001d018571)** | **CCGGATGATGAGGAACAGGG** | **AGGTTCTCATTCACCAGGCG** |
| **ZmABF7(Zm00001d018178)** | **AGGGGTCGGTGTACTCTCTC** | **TGTTCATGGAGCCGAAGTCC** |
| **ZmABF3(Zm00001d042721)** | **TATCGCCGAGCTAGAATCGC** | **CACCCGTTCCAAGAGCTCTT** |

**Continued table S3. Primers for ZmABF gene in qRT PCR experiment**

| **Gene name** | **Forward primer** | **Reserve primer Reserve primer** | | |
| --- | --- | --- | --- | --- |
| **ZmABF5(Zm00001d043117)** | | | **GGACATCCAGGATGCACCAA** | **ACAACCCCAGCTTTCACCAA** |
| **ZmABF12(Zm00001d020711)** | | | **ATTCAAGAACTGCGGGTCGT** | **TTCGTCGAACGTCAACGAGT** |

**Table S4. Primers for ZmABF cloning and plant expression vector construction**

| Gene name | Forward primer | Reserve primer Reserve primer |
| --- | --- | --- |
| ZmABF8 | CCGGATGATGAGGAACAGGG | AGGTTCTCATTCACCAGGCG |
| ZmABF8-3301 | GAGCTCGGTACCCGGGGATCCTCTAGAGTCGACCTGCAGGCATGCAAGCTTATGGGAGACGACGACGACGACGACGAC | TGCATGCCTGCAGGTCGACTCTAGAGGATCCCCGGGTACCGAGCTCTCACTCGAGCTGCAGGTCGACTCTAGA |
| ZmABF8-1302 | GAGCTCGGTACCCGGGGATCCTCTAGAGTCGACCTGCAGGCATGCAAGCTT ATGGGAGACGACGACGACGACGAC | ATGGTGAGCAAGGGCGAGGAGCTGTTCACCGGGGTGGTGCCCATCCTGGTCGAG CTCGAGCTGCAGGTCGACTCTAGA |

**Table S5. Segmental duplications of ABF genes in maize and KaKs ratios analysis.**

| Seq_1 | Seq_2 | Ka | Ks | Ka_Ks | EffectiveLen | AverageS-sites | AverageN-sites | cN | cS | pN | pS | Note |
| --- | --- | --- | --- | --- | --- | --- | --- | --- | --- | --- | --- | --- |
| ZmABF3 | ZmABF11 | 0.2969052213576314 | 1.0640213222612742 | 0.27904066877780603 | 726 | 185.5833333333336 | 540.4166666666664 | 132.5 | 105.49999999999999 | 0.24518118735543573 | 0.5684777727885039 |  |
| ZmABF3 | ZmABF16 | 0.10646532641703264 | 0.17567316896583954 | 0.606042044119642 | 699 | 185.16666666666683 | 513.8333333333331 | 51 | 29 | 0.09925397340253005 | 0.15661566156615647 |  |
| ZmABF4 | ZmABF17 | 0.09426683853225398 | 0.22247601279292073 | 0.4237168643434695 | 1089 | 264.9166666666671 | 824.0833333333328 | 73 | 51 | 0.08858327434523214 | 0.1925133689839569 |  |
| ZmABF5 | ZmABF15 | 0.22501844673762567 | 0.8913942312836394 | 0.2524342640333123 | 954 | 224.83333333333357 | 729.1666666666665 | 141.75 | 117.25 | 0.19440000000000004 | 0.5214974054855444 |  |
| ZmABF7 | ZmABF10 | 0.26014974611997177 | 0.7720268089904155 | 0.33696983458407526 | 978 | 244.08333333333366 | 733.9166666666663 | 161.33333333333334 | 117.66666666666667 | 0.2198251390939027 | 0.4820757937862746 |  |
| ZmABF7 | ZmABF12 | 0.3855523581132783 | 1.3872032168514887 | 0.2779350230951453 | 939 | 235.75000000000034 | 703.2499999999997 | 212 | 148.99999999999997 | 0.3014575186633489 | 0.6320254506892885 |  |
| ZmABF7 | ZmABF18 | 0.24293746003239083 | 0.7937537921476254 | 0.3060614795616729 | 975 | 243.33333333333366 | 731.6666666666663 | 151.83333333333331 | 119.16666666666669 | 0.2075170842824602 | 0.4897260273972597 |  |
| ZmABF11 | ZmABF16 | 0.35415787098865187 | 1.1321331378744515 | 0.3128235179597105 | 648 | 169.16666666666677 | 478.83333333333326 | 135.16666666666669 | 98.83333333333336 | 0.28228332753219637 | 0.5842364532019703 |  |
| ZmABF10 | ZmABF18 | 0.05052529919198597 | 0.1398883397627038 | 0.3611830641331032 | 1008 | 250.75000000000034 | 757.2499999999997 | 37 | 32 | 0.048861010234400815 | 0.1276171485543368 |  |

## Supplementary Figures


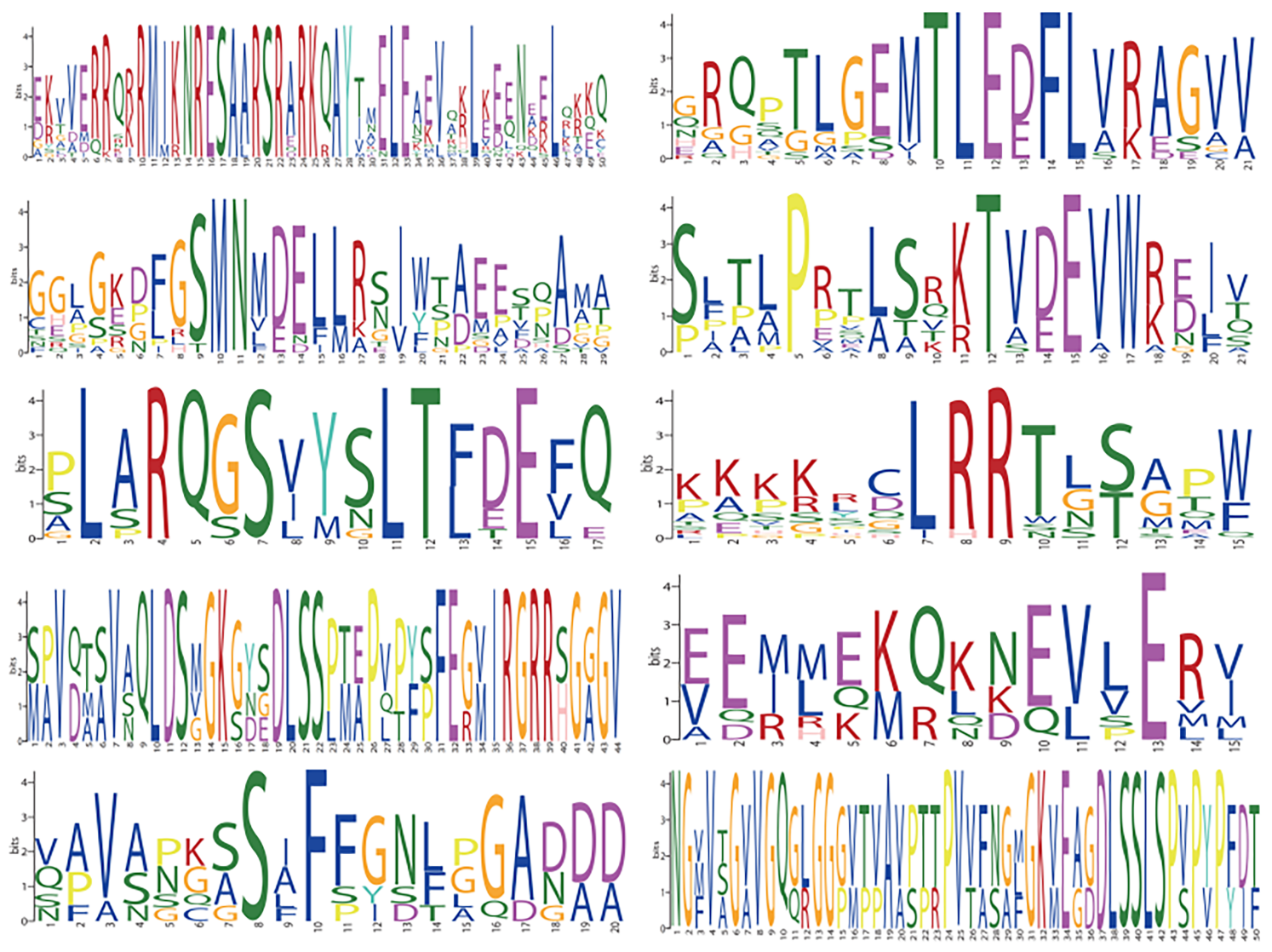


**Fig S1. A conserved motif of the maize ABF gene.**

Motif1-Motif10 represent different conserved motifs, the numbers on the x-axis represent the amino acid position, and the font size represents the relative frequency at the position.
